# Physiological synchrony in heart rate and electrodermal activity predicts decreased vigilant attention induced by sleep deprivation

**DOI:** 10.3389/fnrgo.2023.1199347

**Published:** 2023-06-29

**Authors:** Ivo V. Stuldreher, Emma Maasland, Charelle Bottenheft, Jan B. F. van Erp, Anne-Marie Brouwer

**Affiliations:** ^1^Human Performance, Netherlands Organisation for Applied Scientific Research (TNO), Soesterberg, Netherlands; ^2^Human Media Interaction, Faculty of Electrical Engineering, Mathematics and Computer Science, University of Twente, Enschede, Netherlands; ^3^Human Machine Teaming, Netherlands Organisation for Applied Scientific Research (TNO), Soesterberg, Netherlands; ^4^Donders Institute for Brain, Cognition and Behaviour, Radboud University, Nijmegen, Netherlands

**Keywords:** inter-subject correlation, physiological synchrony, electrodermal activity, heart rate, sleep deprivation

## Abstract

**Introduction:**

When multiple individuals are presented with narrative movie or audio clips, their electrodermal activity (EDA) and heart rate show significant similarities. Higher levels of such inter-subject physiological synchrony are related with higher levels of attention toward the narrative, as for instance expressed by more correctly answered questions about the narrative. We here investigate whether physiological synchrony in EDA and heart rate during watching of movie clips predicts performance on a subsequent vigilant attention task among participants exposed to a night of total sleep deprivation.

**Methods:**

We recorded EDA and heart rate of 54 participants during a night of total sleep deprivation. Every hour from 22:00 to 07:00 participants watched a 10-min movie clip during which we computed inter-subject physiological synchrony. Afterwards, they answered questions about the movie and performed the psychomotor vigilance task (PVT) to capture attentional performance.

**Results:**

We replicated findings that inter-subject correlations in EDA and heart rate predicted the number of correct answers on questions about the movie clips. Furthermore, we found that both inter-subject correlations in EDA and heart rate predicted PVT performance. Individuals' mean EDA and heart rate also predicted their PVT performance, but for both EDA and heart rate, combining mean values with inter-subject correlations yielded better explanation of the PVT performance.

**Discussion:**

Together, these findings confirm the association between physiological synchrony and attention. Physiological synchrony in EDA and heart rate do not only capture the attentional processing during the time that it is determined, but also proves valuable for capturing more general changes in the attentional state of monitored individuals.

## 1. Introduction

When individuals are engaged in social interaction, their physiological signals can align (Palumbo et al., 2017). This similarity in physiological activity across individuals is referred to as physiological synchrony. Physiological synchrony has been shown for diverse groups of socially interacting individuals, such as parent-child dyads (Feldman et al., 2011), therapist-patient dyads (Marci et al., 2007), teammates (Elkins et al., 2009), and pairs of strangers meeting for the first time (Silver and Parente, 2004).

We think that findings of physiological synchrony may be at least partially explained by shared attention. Therefore, we argue that physiological synchrony can be a valuable tool to study shared attention in groups of individuals. In recent years physiological synchrony was indeed shown to reflect shared attention. When individuals attend to a joint narrative stimulus, the inter-subject correlations in the electroencephalogram (EEG), electrodermal activity (EDA), and heart rate are higher than one would expect based on chance (Poulsen et al., 2017; Stuldreher et al., 2023b). Altering the attentional focus of individuals was found to affect inter-subject correlations. Individuals showed decreased physiological synchrony when instructed to focus attention inward on a mental arithmetic task instead of the joint stimulus (Ki et al., 2016; Pérez et al., 2021). Individuals instructed to focus only on specific stimulus parts showed higher inter-subject correlations with individuals instructed to focus on the same instead of on different stimulus parts (Stuldreher et al., 2020b). Individuals with higher participant-to-group inter-subject correlations in EEG or heart rate better recalled the narrative (Cohen and Parra, 2016; Stuldreher et al., 2020b). In sum, physiological synchrony can be altered by interventions on attention and has been found to be associated with performance on a task that reflects how well a presented narrative was attended to.

There are also indications that inter-subject correlations in EEG and functional magnetic resonance imaging can capture interpersonal variations in attentional processing that relate to personality traits. For instance, we found that food neophobia, the hesitance to try new foods, was positively correlated with inter-subject correlations in EEG during a movie about a foreign food (Stuldreher et al., 2023a). That is, individuals who scored higher on the food neophobia scale showed less inter-subject correlations in EEG. In addition, individuals with autism spectrum disorders, depression or first-episode psychosis, are known to show more varying neural patterns and thus reduced neural inter-subject correlations during naturalistic stimuli than typically developing individuals (Hasson et al., 2009; Salmi et al., 2013; Guo et al., 2015; Mäntylä et al., 2018).

The indications that variations in attentional processing within an individual can also be captured by inter-subject correlations are limited. It is established that inter-subject correlations in EEG decrease when viewing the same stimulus for the second time (Dmochowski et al., 2012; Ki et al., 2016), consistent with participants being less interested in the stimulus upon a second viewing. We may expect that inter-subject correlations as established during a short narrative can capture the momentary attentional state of an individual, and therefore predict performance on subsequent attentional tasks. However, inter-subject correlations have never been related to performance on attentional tasks separate from the narrative. As of yet, it is not clear whether inter-subject correlations monitored during a narrative presentation can capture variations in attentional processing abilities as also reflected in another task.

In the current work, we manipulate the attentional abilities within individuals over time by exposing them to a night of total sleep deprivation. During the night, we monitor inter-subject correlations in EDA and heart rate during the presentation of movie clips. Although work on inter-subject correlation as measure of attention originated in measures of brain activity such as functional magnetic resonance imaging (e.g., Hasson et al., 2004, 2008) and EEG (e.g., Dmochowski et al., 2012), we and others found that inter-subject correlations in body measures such as EDA and heart rate reflect attention as well (Stuldreher et al., 2020a,b; Pérez et al., 2021; Madsen and Parra, 2022). This also holds when using measurements from wearables (Van Beers et al., 2020).

Sleep deprivation is known to have strong detrimental effects on attention, working memory and decision making (Alhola and Polo-Kantola, 2007; Pilcher et al., 2007). It especially impacts cognitive functioning in long, simple and monotonous tasks requiring reaction speed or vigilance (Alhola and Polo-Kantola, 2007; Lim and Dinges, 2008; Hudson et al., 2020). Pilcher et al. (2007) provide a reasoning for the especially strong effects of sleep deprivation on undemanding tasks through their model of attentional control. Undemanding cognitive tasks, like vigilance tasks, require more internal control over one's attention, since there are less external stimuli to keep one engaged. Sleep deprived individuals are thought to have difficulty exerting this internal control over their attention. We investigate whether inter-subject correlations in EDA and heart rate can predict performance on a task demanding vigilant attention, i.e., a task that is expected to be strongly affected by sleep deprivation. A positive result in this study would indicate that physiological synchrony may indeed capture the varying attentional abilities of individuals and would extend the predictive value of physiological synchrony on attentional performance to moments beyond the time that physiological synchrony was determined. Instead of investigating whether inter-subject correlations vary between discrete attentional conditions, this study allows us to explore the predictive value of inter-subject correlations on differences in attention that are of more continuous nature.

We compare the predictive value of inter-subject correlations in EDA and heart rate to the predictive value of individuals' mean EDA and heart rate. EDA and heart rate on individual level are known to be affected by sleep deprivation, and have been associated with cognitive performance during a sleep deprived night before (Miró et al., 2002; Posada-Quintero et al., 2017). Directly comparing the predictive value of inter-subject correlations with the individual physiological features allows us to display potential added value of inter-subject analyses.

In sum, we aim to answer the following main research question:

1. How well does physiological synchrony in EDA and heart rate predict later vigilant attention during a night of sleep deprivation?

Additionally, we aim to answer the following secondary research question:

2. How does the predictive value of physiological synchrony relate to predictive value of individual's mean physiological activity?

## 2. Materials and methods

### 2.1. Participants

One hundred and one Dutch speaking volunteers took part in a 2-day study on the effects of sleep deprivation on cognitive performance. They were randomly assigned to the sleep deprivation condition (*N* = 54) or control condition (*N* = 48). This study describes results obtained from participants in sleep deprivation condition during the night. These 54 participants (29 female) were between 18 and 55 years old (*M* = 29.4, *SD* = 11.9). The study was approved by the medical research ethics committee (MREC) Brabant (reference number: P2045, approval number: NL74961.028.20). All participants provided written informed consent before partaking in the experiment. Exclusion criteria were: smoking, drug use in the last 3 months, signs of flue or viral infection in the last 10 days, pregnant, history of psychiatric illness, including sleep disorders, autoimmune disease and/or hyperactive thyroid and people with known heart, kidney or liver disease, or neurological complaints.

### 2.2. Physiological measurements

Participants' EDA and heart rate were recorded throughout the night. EDA was recorded at 32 Hz with an EdaMove 4 (Movisens GmbH, Karlsruhe, Germany), that was recording signals from the palmar surface of the non-dominant hand using two solid gelled Ag/AgCl electrodes (MTG IMIELLA electrode, MTG Medizintechnik, Lugau, Germany, W55 SG, textured fleece electrodes, 55 mm diameter). Heart rate was recorded at 1 Hz and with 1 bpm resolution using a Tickr chest-strap (Wahoo Fitness, Atlanta, GA, USA) that was coupled to an Android smartphone (Samsung Galaxy A41, Android version 10) via Bluetooth. Data were received, processed and saved with the use of the Wahoo Fitness Workout Application (version 1.40.0.56).

### 2.3. Stimuli

#### 2.3.1. Movie clips

Over the course of the night, participants were presented with ten 10-min movie clips (M = 9:56, min. = 9:04, max. = 10:58) every hour from 22:00 to 07:00. The movie clips were selected from the Dutch YouTube channels NPO3 and KORT! and featured short, moderately engaging stories. In a previous study using six of these movies we showed that they were effective in eliciting significant inter-subject correlations in EDA and heart rate (Stuldreher et al., 2023b). The order of presentation was the same for each participant. This presentation order, movie duration and URL for each movie can be found in Table 1.

**Table 1:** Details and order of the presented movie clips.

Order	Name	Duration (min)	URL
1	Chauffeur	09:45	https://www.youtube.com/watch?v=jaFmvyH7dW8
2	El Mourabbi	09:04	https://www.youtube.com/watch?v=X9bJou2gKxo
3	De Chinese Muur	09:50	https://www.youtube.com/watch?v=yjGFuhPy3Qo
4	One of the boys	10:58	https://www.youtube.com/watch?v=PsGAuhgQ97k
5	Samual	09:45	https://www.youtube.com/watch?v=VUseoqCVnj4
6	Turn it around	09:26	https://www.youtube.com/watch?v=beC7IpQpTz4
7	En route	10:05	https://www.youtube.com/watch?v=M6ebApnH_XE
8	Mowgli en Fidel	10:03	https://www.youtube.com/watch?v=MocrSQW_r_M
9	Heen en weer dag	10:25	https://www.youtube.com/watch?v=yPueHzj9STE
10	Gutmensch	10:02	https://www.youtube.com/watch?v=P7MABwTYa58

Directly after each movie, participants answered 10 multiple-choice questions (four answers of which one correct). The questions concerned both general and specific details of the movie. The questions and answer options can be found in the Supplementary Table 1.

#### 2.3.2. Psychomotor vigilance task (PVT)

After every sequence of watching a movie and answering the questions, participants performed the psychomotor vigilance task (PVT). The experimental software that presented the PVT was custom-made for this experiment using Python (version 3.8) and the compatible PsychoPy toolbox (Peirce et al., 2019). The PVT was designed to be maximally sensitive to the effects of sleep deprivation by following recommendations from Basner and Dinges (2011). The course of the PVT was as follows. After pressing the spacebar to initialize the task, a black square (9.5° of visual angle) surrounded by a red border centered in the middle of a gray screen was shown. For each trial, a random interval between 2 and 10 seconds was selected, after which a counter appeared in the center of the black square. The counter consisted of three yellow digits displaying the milliseconds since the onset of the counter. The participants' task was to press spacebar as soon as possible after appearance of the counter. As soon as they did, the reaction time was displayed for 2 s. If participants failed to press the spacebar within 3 s of the counter appearing or if participants pressed the spacebar without a counter being present, a yellow “X” appeared in the black square instead of the digits. This error message stayed present for 2 s, after which the black square was cleared again and a new trial started. The PVT lasted 10 min, and the amount of trials varied based on the reaction times and misses of each participant.

### 2.4. Procedure

Data from participants was collected as part of a larger study over the course of seven experimental sessions conducted in 2021 between March and June. Each experimental session consisted of two morning sessions that are discussed in more detail in Bottenheft et al. (submitted), and the night spent at the research institute between the two mornings in which the measurements for this study were performed. In each experimental session on average 7.7 participants (SD = 1.48) participated. Participants joined a training session 2 weeks before the experimental session. During the training session, participants were familiarized with tasks they would have to perform during the course of the experiment. Of relevance for the current study is that the participants familiarized themselves with the PVT for 5 min. Additionally, they were taught how to self-apply the Tickr chest strap and EdaMove 4 (a safety measure taken to reduce the risk of spreading the COVID-19 virus). Furthermore, participants received instructions not to consume any caffeinated substance after 18:00 on the day of their experimental session and not to consume any alcohol in the 24 h prior to the experiment.

**Figure 1:**
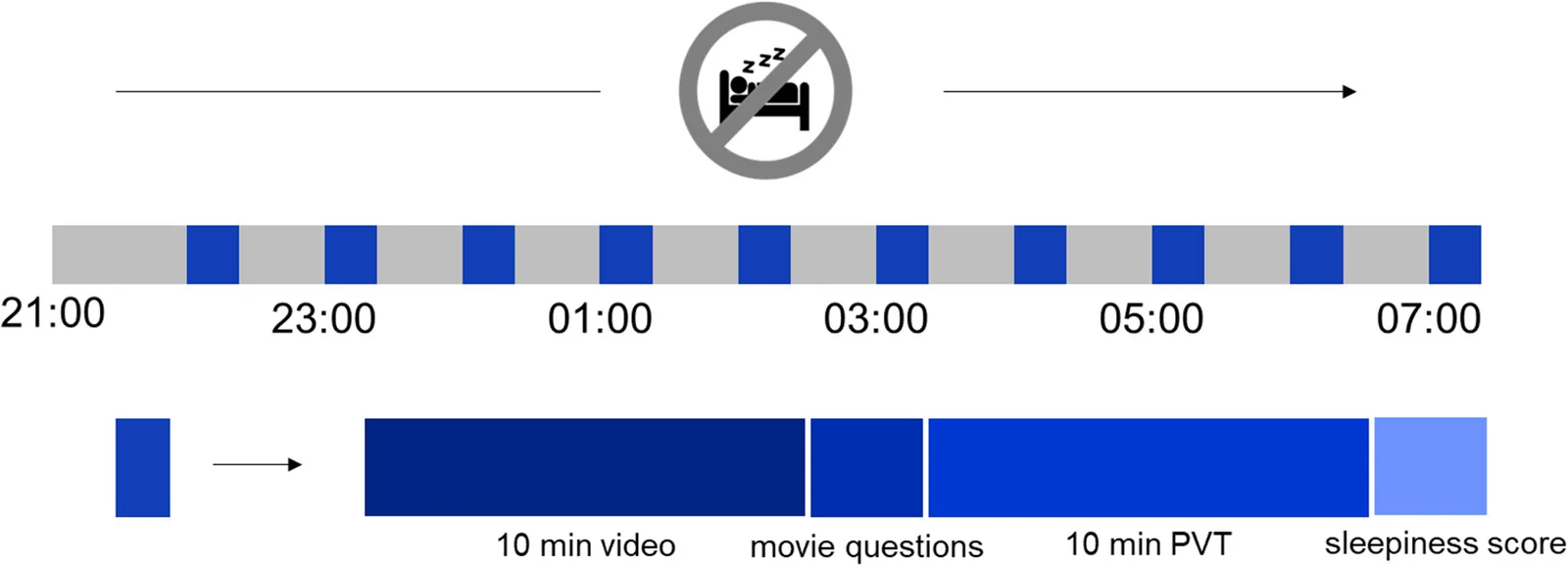
The experiment consisted of 10 blocks of a 10 min movie clip, followed by questions about the movie, a 10 min vigilance task and the Stanford sleepiness scale. These blocks started each hour from 22:00 to 07:00 o'clock.

On the day of the night session, participants came to the research institute at 21:00. Figure 1 describes the full procedure of the night session. Participants were led to the cafeteria of the research institute, where they spent most of the time during the night. Participants were instructed to apply the Wahoo Tickr and start a new measuring session in the Wahoo Fitness Workout Tracker application on the smart phone they had received for the duration of the study. Participants also applied the EdaMove 4 and corresponding electrodes. A researcher checked whether the data recording of both devices was running. After this procedure, participants were given a 30 min rest period. During this and other resting periods, participants were allowed to move around freely and talk to one another or entertain themselves with a book, game or electronic device they brought from home. They were not allowed to exercise. If a participant fell asleep the researcher woke the participant.

During the subsequent time of the experimental session, participants followed a standard procedure every hour, depicted in Figure 1. The procedure consisted of the following elements: watching a 10-min movie clip and answering 10 questions about the presented movie, performing a 10-min PVT and reporting their sleepiness on the Stanford Sleepiness Scale (SSS). After they had completed the procedure (about 30 min), participants were allowed to rest until the initiation of the next cycle. The first cycle was initiated at 22:00, and every subsequent cycle was initiated on the following hour. The last cycle was performed at 07:00. At 23:30, 01:30, and 04:30, participants received a snack. They received 150 g of full fat quark, 150 g of fresh vegetables with hummus, or 30 g of walnuts and could pick which snack they wanted at which point in time. They were allowed to drink water and theine-free teas.

### 2.5. Analysis

Data were analyzed using MATLAB R2021a (Mathworks, Natick, MA, USA). The data and MATLAB scripts used are available online at: https://osf.io/69u8h/.

To be able to directly compare the predictive results of inter-subject correlations in EDA and heart rate, we used only data of participants for which EDA and heart rate recordings were available. Heart rate data of six participants were unavailable due to lost data recordings. EDA data of an additional two participants were lost due to sensor failure. For 10 additional participants, EDA or heart rate data during part of the recording blocks were lost due to detached electrodes or disconnected Bluetooth connection. In total 21 recording blocks were lost across these participants. In sum, the following analyses are conducted using 46 participants, with in total 439 recording blocks.

#### 2.5.1. PVT analysis

PVT response times were processed to obtain the lapse probability. A PVT response was considered valid if given between 100 ms and 3,000 ms after stimulus onsets. Responses within 100 ms of stimulus onset and responses without a stimulus being present were considered false alarms. Responses that occurred more than 500 ms after stimulus onset were considered lapses (Basner and Dinges, 2011). The lapse probability was computed by dividing the amount of lapses with the amount of correct responses excluding lapses. Invalid responses are thus not considered in the lapse probability.

#### 2.5.2. EDA and heart rate

EDA was resampled to 8 Hz. EDA and heart rate were epoched to the on- and offset of each movie using markers sent by the experimental program. EDA was then further processed to obtain the phasic component, also called skin conductance response (SCR), as this component of EDA is characterized by fast consecutive response-like changes. To do so, we used continuous decomposition analysis as implemented in the Ledalab toolbox for MATLAB (Benedek and Kaernbach, 2010). We use the SCR, as external stimuli mainly affect this component of EDA.

The average SCR and heart rate over each epoch were used as input for the predictive models that use individuals' physiological signals as predictor, as described below. The time-course of the SCR and heart rate were used for computation of inter-subject correlations. In the following parts of the manuscript, when we use the term EDA we refer to the SCR component of the electrodermal signal.

#### 2.5.3. Inter-subject correlations

Physiological synchrony was quantified with the use of inter-subject correlations. For each participant's EDA and heart rate during a given movie, inter-subject correlations were computed with the EDA and heart rate of every other participant during that same movie, following our previous procedure (Stuldreher et al., 2023b). When averaging over the inter-subject correlations with all other participants, we obtain a metric we refer to as participant-to-group inter-subject correlation. This metric is used as the predictor variable. From here on, if we refer to inter-subject correlation we refer to this participant-to-group metric.

To test the significance of participant-to-group inter-subject correlation values over chance level, we used the circular shuffle statistic, following (Pérez et al., 2021; Madsen and Parra, 2022). Each participant's physiological signal during each movie was circular shifted by a random amount within the epoch length. The inter-subject correlations and participant-to-group inter-subject correlations were then computed with this circular shuffled data. This procedure was repeated 500 times for each participant and each movie to estimate the chance distribution of inter-subject correlations. Note that a higher number of shuffles (e.g., 10.000 as in Pérez et al., 2021; Madsen and Parra, 2022) would result in a better estimation of the chance distribution. The *p*-value then is the fraction of circular shuffles with inter-subject correlation values higher than the original unshuffled inter-subject correlations.

As an additional check, we computed participant-to-group inter-subject correlation values using data from non-matching movies to compare the real value to. That is, instead of computing inter-subject correlations of participant *x* with participant *y* using data from the same movie *m*_1_, we computed inter-subject correlations between data of participant *x* from movie *m*_1_ with data of participant *y* from all movie clips *m*_2_ in *M*_2_, where *M*_2_ are all videos not equal to *m*_1_. For the most stringent test, we then selected the maximum inter-subject correlation value to compare to the real inter-subject correlation values. Comparisons were done using paired sample *t*-tests.

Note that we do not necessarily expect inter-subject correlations higher than chance level for all times at night. We expect low, or even absent inter-subject correlations if participants do not attend to the movies because of sleepiness.

#### 2.5.4. Hierarchical linear analysis

We used an hierarchical linear model (HLM) for the main statistical analysis. An HLM was selected since the data is organized hierarchically and clustered within individuals. Therefore, the assumption of independent observations required for a regular regression is violated.

We performed the hierarchical linear regression in four steps, shown in Table 2. In the first step, that serves as a baseline, only the dependent variable is added to the model. In the second step, the level two variable individual is added to the model, to investigate if allowing the means of the dependent variable to vary across individuals leads to improvement of the model. In the third step, the predictor is added with a fixed slope and random intercept. This step allowed us to investigate whether the predictor has a main predictive effect on the dependent variable. In the fourth and final step, the predictor variable is added with a random slope and random intercept, meaning that the model allows the relationship between the predictor and the dependent variable to vary between individuals.

**Table 2:** Steps of the hierarchical linear model analysis.

Step	Formula
1	*V*_*dep*_ ~ 1
2	*V*_*dep*_ ~ 1 + (1 | participant)
3	*V*_*dep*_ ~*p*_1_ + (1 | participant)
4	*V*_*dep*_ ~*p*_1_ + (*p*_1_ | participant)

*V*_*dep*_, dependent variable, either log transformed PVT lapse probability scores or number of correctly answered on questions about the movies; *p*_1_, predictor variable 1, either inter-subject correlations in EDA or heart rate, mean EDA or heart rate.

We first built models with number of correct answers on questions about the movie as dependent variable, individual as level two variable and inter-subject correlations in EDA or heart rate as predictor. This was done to investigate whether the previously found relation between narrative retention and inter-subject correlations could be replicated in the current setting.

We then built models with PVT lapse probability as a dependent variable, individual as a level two variable and inter-subject correlations in EDA or heart rate as predictor. This analysis was conducted to answer the main research question, to explore the potential association between inter-subject correlation and performance on a subsequent task requiring a different form of attention.

Then, we used individuals' mean EDA or heart rate as predictor of PVT lapse probability. This was done to compare the predictive value of inter-subject correlations to individuals' physiological activation.

Before performing the HLM analyses, inter-subject correlation scores were centered using grand-mean sampling to avoid collinearity problems. PVT lapse probability was log transformed as untransformed data were positively skewed.

## 3. Results

### 3.1. Significance of inter-subject correlations as measure of attentional engagement

**Figure 2:**
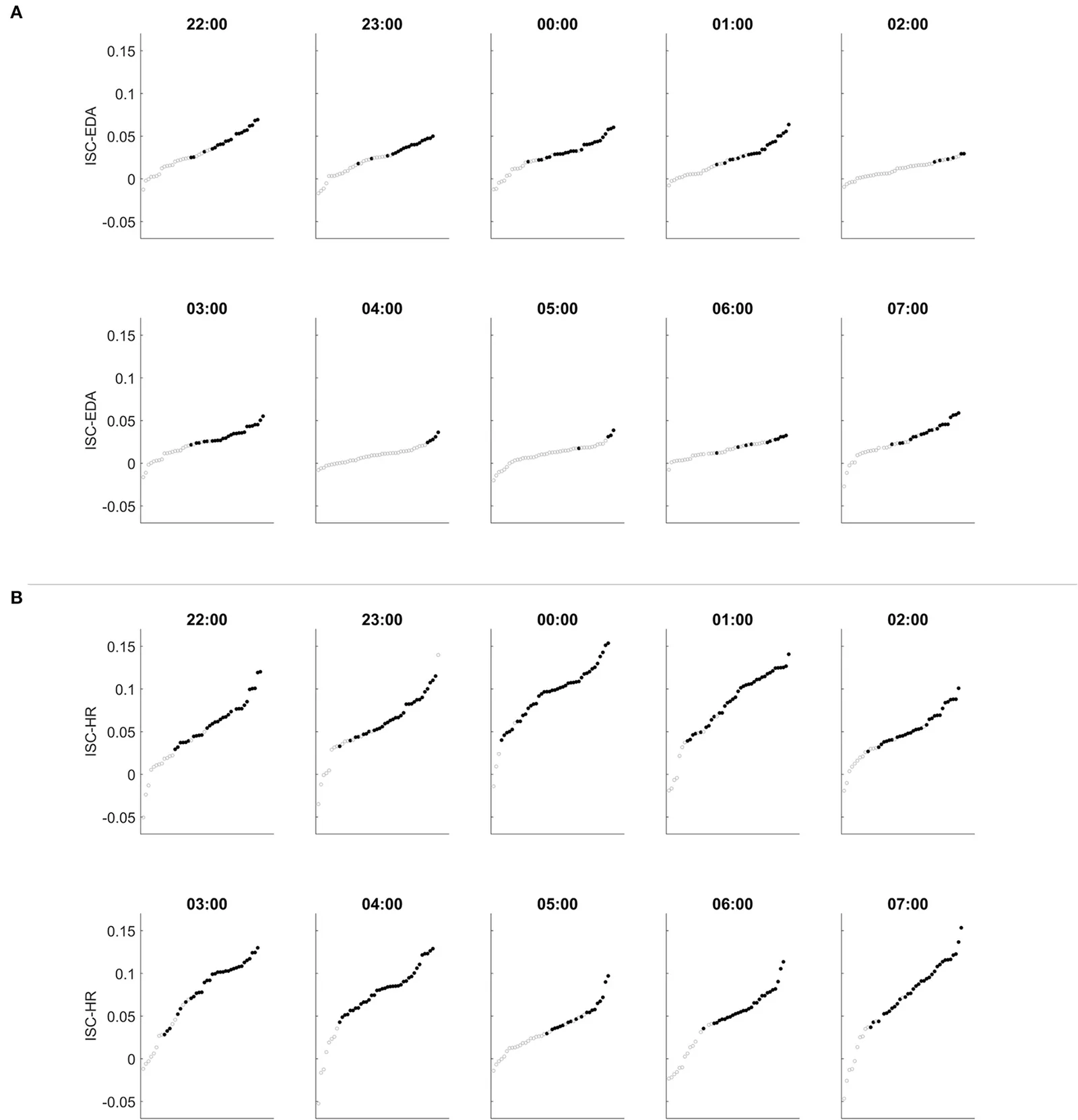
Inter-subject correlations in EDA (**A**; ISC-EDA) and heart rate (**B**; ISC-HR) for each individual and each movie clip ordered from low to high. Each marker refers to the participant-to-group inter-subject correlations of a participant. Filled markers depict inter-subject correlations significantly higher than chance, open markers depict inter-subject correlations not higher than chance level.

**Figure 3:**
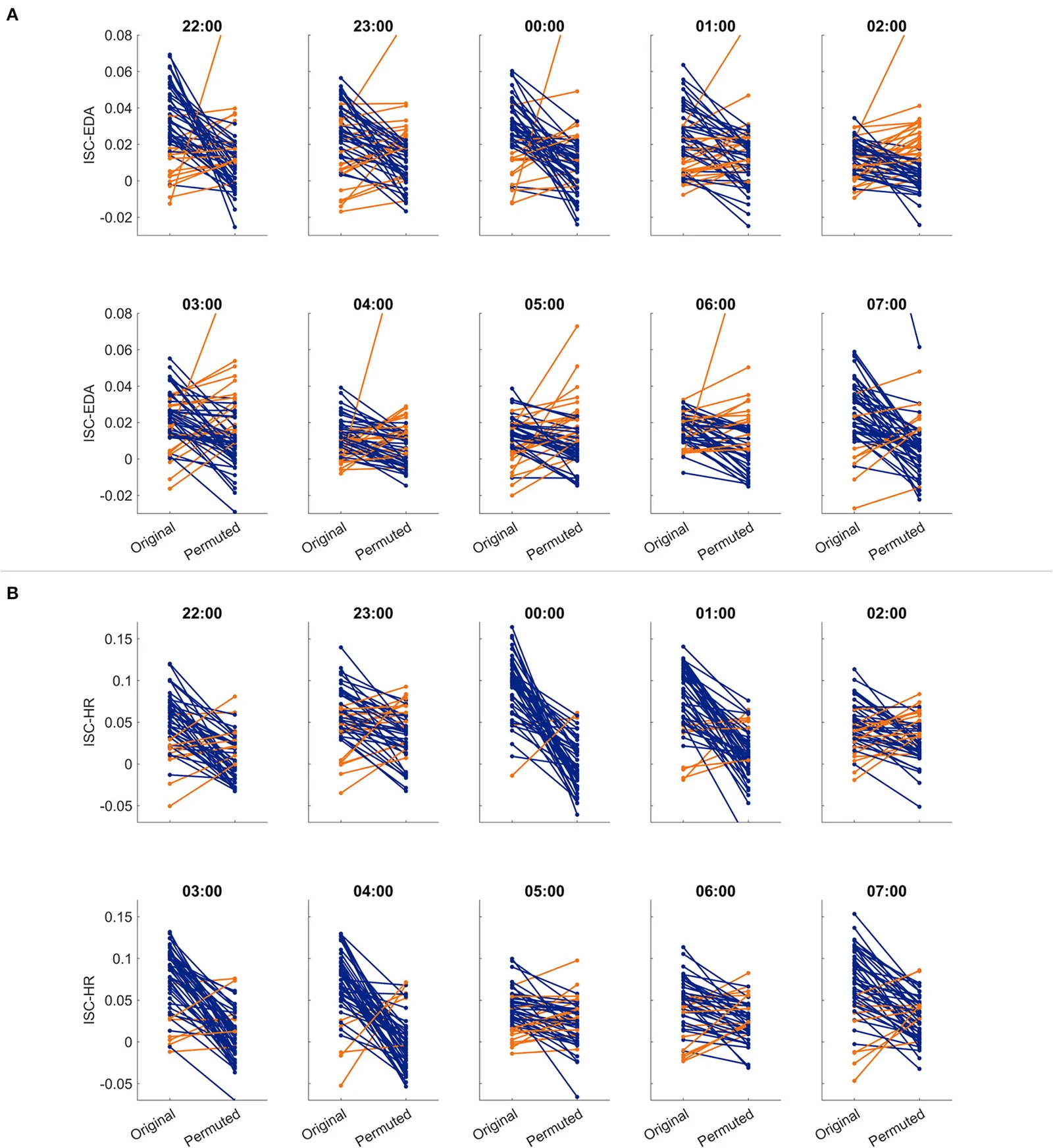
Inter-subject correlations in EDA (**A**; ISC-EDA) and heart rate (**B**; ISC-HR) for each individual and each movie compared to a permuted inter-subject correlations obtained by not matching the movies between participants. Blue lines depict participants where the permuted inter-subject correlations are lower than the original values, orange lines depict participants where the permuted inter-subject correlations are higher than the original values.

Before exploring potential associations between inter-subject correlations and attention, we establish to what extent the inter-subject correlations are higher than one would expect based on chance using two approaches. First, we investigated whether real inter-subject correlations were higher than inter-subject correlations obtained from circular shuffled data. Figure 2 depicts the inter-subject correlations in EDA and heart rate for each individual and each movie clip ordered from low to high. In each panel, each marker refers to a single participant. Filled markers depict inter-subject correlations that are significantly higher than chance level, open markers depict inter-subject correlations that are not significantly higher than chance level. For heart rate, during each movie on average 73.2% (SD: 12.3%) of the participants show significant inter-subject correlations. In EDA, during each movie on average 36.6% (SD: 18.8%) of the participants show significant inter-subject correlations. Supplementary Figure 1 shows the same inter-subject correlations and compares these to the chance level distributions obtained through 500 instances of circular shuffling. We then investigated whether real inter-subject correlations where higher than inter-subject correlations obtained when movies did not match between participants. Figure 3 depicts the inter-subject correlations in EDA and heart rate for each individual and each movie and compares these to inter-subject correlations obtained after a permutation in which non-matching movies were used. In each panel each line refers to data of a single participant. Blue lines depict participants where the permuted inter-subject correlations are lower than the original values, orange lines depict participants where the permuted inter-subject correlations are higher than the original values. A set of paired sample *t*-tests showed that the real inter-subject correlation values were significantly higher than the permuted ones in most cases. The *t*-test statistics can be found in Supplementary Table 2.

### 3.2. Inter-subject correlations as predictor of number of correctly answered questions about the movie

**Figure 4:**
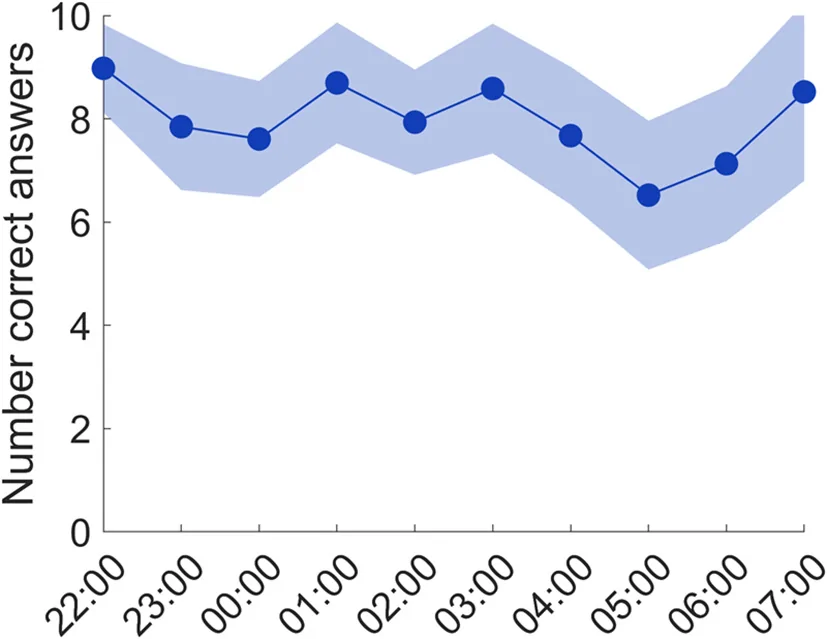
Number of correct answers to questions about the content of the movie over the course of the night. Markers depict the mean across participants, shaded area depicts the standard deviation.

Our next step was to check whether we could replicate previous reports of an association between inter-subject correlations and the number of correctly answered questions about the presented movie clips. Figure 4 shows how the number of correct answers varies over the course of the night. It suggests a slight drop in the number of correct answers up to 05:00 o'clock, followed by an increase up to 07:00 o'clock. Table 3 shows the statistical parameters of the HLM analysis used to identify a potential association between inter-subject correlations and the number of correct answers. For each step, the table displays the minus 2 Log Likelihood statistic (−2LL), degrees of freedom (DF), and the Akaike Information Criterion (AIC). The −2LL is a statistic that can be used to compare whether a model is a significant improvement of another model (Field, 2013). The AIC is a more complex measure that can also be used to compare models. The model that best fits the data will display lowest AIC. The table also shows the Chi-square (χ^2^) −2LL change statistic, that describes whether the change in −2LL is significantly different from the previous model. Also displayed is the percentage of total explained variance and the *t*-statistics of the fixed effects.

**Table 3:** Statistical parameters of the two hierarchical linear models using inter-subject correlations in either EDA (ISC-EDA) or heart rate (ISC-HR) as predictor of the number of correct answers about the content of the movies.

Step	Predictor	−2LL	DF	AIC	χ^2^ −2LL change	*R* ^2^	t fixed effect
1		−794.76	2	1,593.2			
2		−794.76	3	1,595.5	(1, *N* = 439) = 0, *p* = 1	0	112.48
3a	ISC-EDA	−791.89	4	1,591.8	(1, *N* = 439) = 5.75, *p* = 0.016	0.013	2.41^*^
3b	ISC-HR	−783.74	4	1,575.5	(1, *N* = 439) = 22.04, *p* < 0.001	0.049	4.75^***^
4a	ISC-EDA	−783.82	6	1,579.6	(2, *N* = 439) = 16.15, *p* < 0.001	0.084	2.70^**^
4b	ISC-HR	−779.5	6	1,571.1	(2, *N* = 439) = 8.40, *p* = 0.015	0.088	4.03^***^

^*^p < 0.05.

^**^p < 0.01.

^***^p < 0.001.

−2LL, minus 2 Log Likelihood statistic; DF, degrees of freedom; AIC, Akaike Information Criterion.

In step one, only the dependent variable (i.e., the number of correct answers) was added to the model to serve as a baseline. The AIC is in this model is 1,593.5.

In step two, the level two variable participant was added to the model to investigate whether allowing for individual differences improves the model prediction. The AIC is in this model is 1,595.5, and the model did not significantly improve compared to step one, χ^2^(1, *N* = 439) = 0, *p* = 1.

In step three, the predictor inter-subject correlations in either EDA (step 3a) or heart rate (step 3b) was added to the model with fixed slope and random intercept. Both for EDA, χ^2^(1, *N* = 439) = 5.75, *p* = 0.016, and heart rate, χ^2^(1, *N* = 439) = 22.04, *p* < 0.001, the prediction significantly improved compared to model step 2. Inter-subject correlations thus have a main predictive effect of the number of correctly answered questions about the movie clips, both for EDA (β = 0.17, *p* = 0.017) and heart rate (β = 0.33, *p* < 0.001).

In step four, the predictor was added with random slope and random intercept, meaning that the model allows the relationship between the predictor and dependent variable to vary across participants. The prediction significantly improved compared to step three, both for EDA (step 4a), χ^2^(2, *N* = 439) = 16.15, *p* < 0.001, and heart rate (step 4b), χ^2^(2, *N* = 439) = 8.40, *p* = 0.015. This indicates that the specific association between inter-subject correlations and number of correct answers is individual specific.

We repeated the four steps of the analysis using the permuted inter-subject correlation values obtained after using non-matching movies. If physiological synchrony indeed reflects the level of attention to the same presented stimulus, no association between the permuted inter-subject correlation values and the number of correct answers is expected. Indeed, there was no significant main predictive effect of the permuted inter-subject correlation values of EDA and heart rate on the number of correct answers on the content of the movie. The statistical parameters of the HLMs used for this analysis can be found in Supplementary Table 3.

### 3.3. Inter-subject correlations in EDA or heart rate as predictor of vigilance

**Figure 5:**
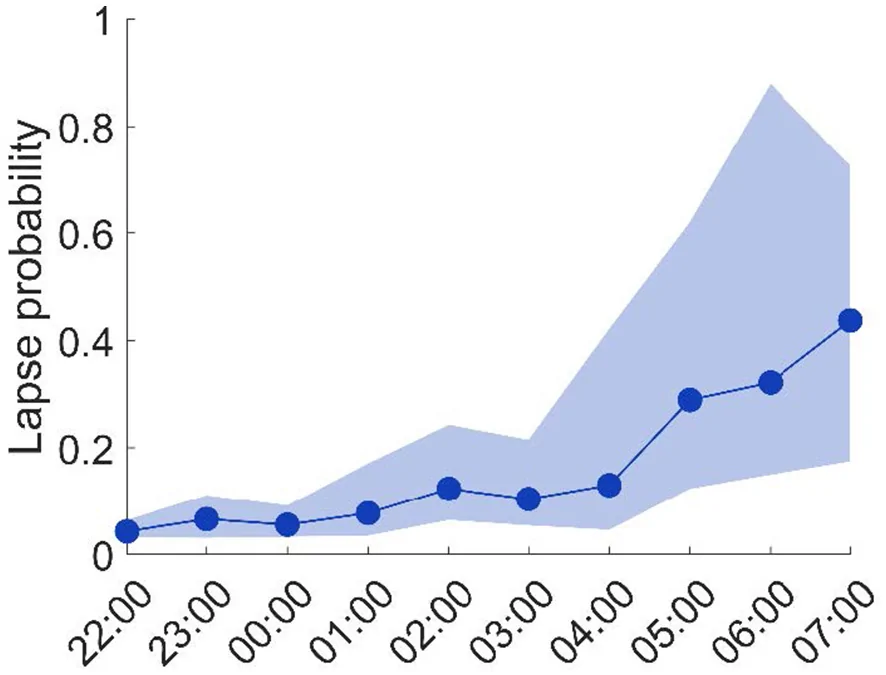
PVT lapse probability over the course of the night. The markers depict the median across participants, shaded area depict the 25–75th percentile.

Next, we investigated the potential association between inter-subject correlation in EDA and heart rate and performance on a subsequent attentional task, i.e., the PVT. A premise for our analysis is that vigilant performance is affected by sleep deprivation and thus varies over the course of the night. Figure 5 shows the PVT lapse probability over the course of the night. The median lapse probability gradually increases from close to zero up to 0.4 at 07:00 o'clock. Consistent with previous findings are the large individual differences in vigilant performance during the night (Hudson et al., 2020), indicated by the shaded area in the figure.

**Figure 6:**
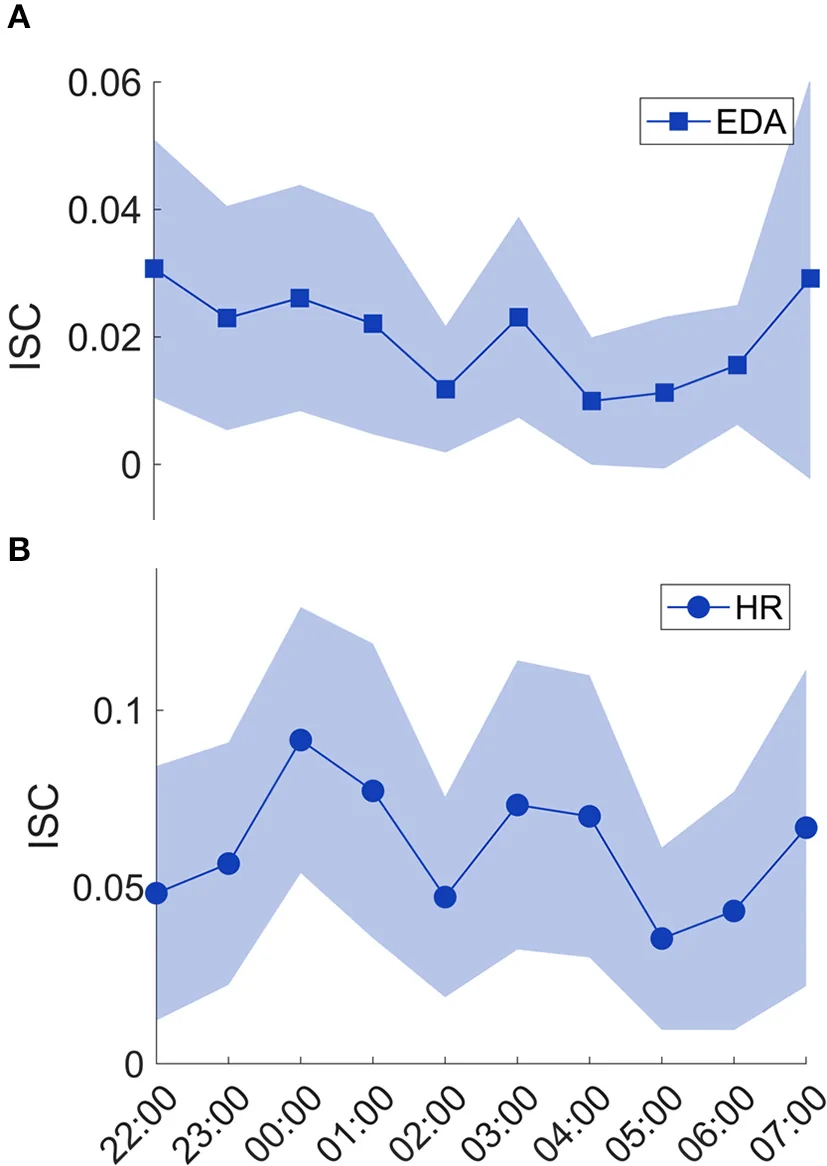
Inter-subject correlations in EDA **(A)** and heart rate (HR; **B**) over the course of the night. Markers depict the mean across participants, shaded area depicts the standard deviation.

A second premise for inter-subject correlations to be predictive of vigilant performance throughout the night is that the intersubject correlations vary throughout the night. Figure 6 shows inter-subject correlations in EDA and heart rate over the course of the night. Inter-subject correlations in heart rate do not seem to follow a consistent pattern throughout the night. Inter-subject correlations in EDA seem more consistent with the overall lapse probability. Except for the last datapoint at 7:00, the figure suggests a decreasing trend of inter-subject correlations in EDA.

Table 4 summarizes the results of the four steps of HLM analyses used to investigate how well inter-subject correlations in either EDA or heart rate predict PVT lapse probability. In step one, only the dependent variable PVT lapse probability was added to the model to serve as a baseline. The AIC in this model is 700.12.

**Table 4:** Statistical parameters of the two hierarchical linear models using inter-subject correlations in either EDA (ISC-EDA) or heart rate (ISC-HR) as predictor of PVT lapse probability.

Step	Predictor	−2LL	DF	AIC	χ^2^ – 2LL change	*R* ^2^	*t* fixed effect
1		−348.06	2	700.12			
2		−287.44	3	580.87	(1, *N* = 438) = 121.25, *p* < 0.001	0.365	–
3a	ISC-EDA	−284.23	4	576.46	(1, *N* = 438) = 6.41, *p* = 0.011	0.379	−2.55^*^
3b	ISC-HR	−280.87	4	569.74	(1, *N* = 438) = 13.14, *p* < 0.001	0.386	−3.65^***^
4a	ISC-EDA	−281.60	6	575.20	(1, *N* = 438) = 5.27, *p* = 0.072	0.396	−2.62^**^
4b	ISC-HR	−280.47	6	572.94	(1, *N* = 438) = 0.80, *p* = 0.670	0.405	−3.42^***^

^*^p < 0.05. ^**^p < 0.01. ^***^p < 0.001. −2LL, minus 2 Log Likelihood statistic; DF, degrees of freedom; AIC, Akaike Information Criterion.

In step two, the level two variable participant was added to the model to investigate whether allowing for individual differences improves the model prediction. The AIC in this model is 580.87. There is a highly significant change in the −2LL, χ^2^(1, *N* = 438) = 121.21, *p* < 0.001. This step of the model explains 36.5% of the variance in PVT lapse probability. The fixed effect of individual on PVT lapse probability is highly significant (*p* < 0.001).

In step three, the predictor, being inter-subject correlations in either EDA (step 3a) or heart rate (step 3b), was added to the model with a fixed slope and random intercept. The results in this step indicate whether the predictor has a main predictive effect on PVT lapse probability. Adding the predictor with a fixed slope means that the model assumes that for all individuals, the relationship between the predictor and PVT lapse probability is identical (Grinsted et al., 2004). In this step, the AIC value is 576.46 for EDA and 569.74 for heart rate. The −2LL value for EDA is −284.23, a significant improvement compared to the previous step, χ^2^(1, *N* = 438) = 6.41, *p* = 0.011. The total variance explained is 37.9% and the fixed effect is significant (*p* < 0.05). For heart rate, the −2LL value (−280.87) is also significantly lower than the model in step two, χ^2^(1, *N* = 438) = 13.14, *p* < 0.001. The total variance explained is 38.6% and fixed effect is highly significant (*p* < 0.001).

In step four, the predictor variable was added with a random slope and a random intercept, meaning that the model allows the relationship between inter-subject correlations and PVT lapse probabilities to vary between individuals. Neither for EDA (step 4a) nor for heart rate (step 4b) the model in this step was significantly better than the model in step three (EDA: AIC = 575.20, −2LL = −281.60, −2LL χ^2^ (2, *N* = 438) = 05.27, *p* = 0.072; heart rate: AIC = 572.94, −2LL = −280.47, −2LL χ^2^ (2, *N* = 438) = 0.80, *p* = 0.670).

### 3.4. Individuals' mean EDA or heart rate as predictor of vigilance

**Figure 7:**
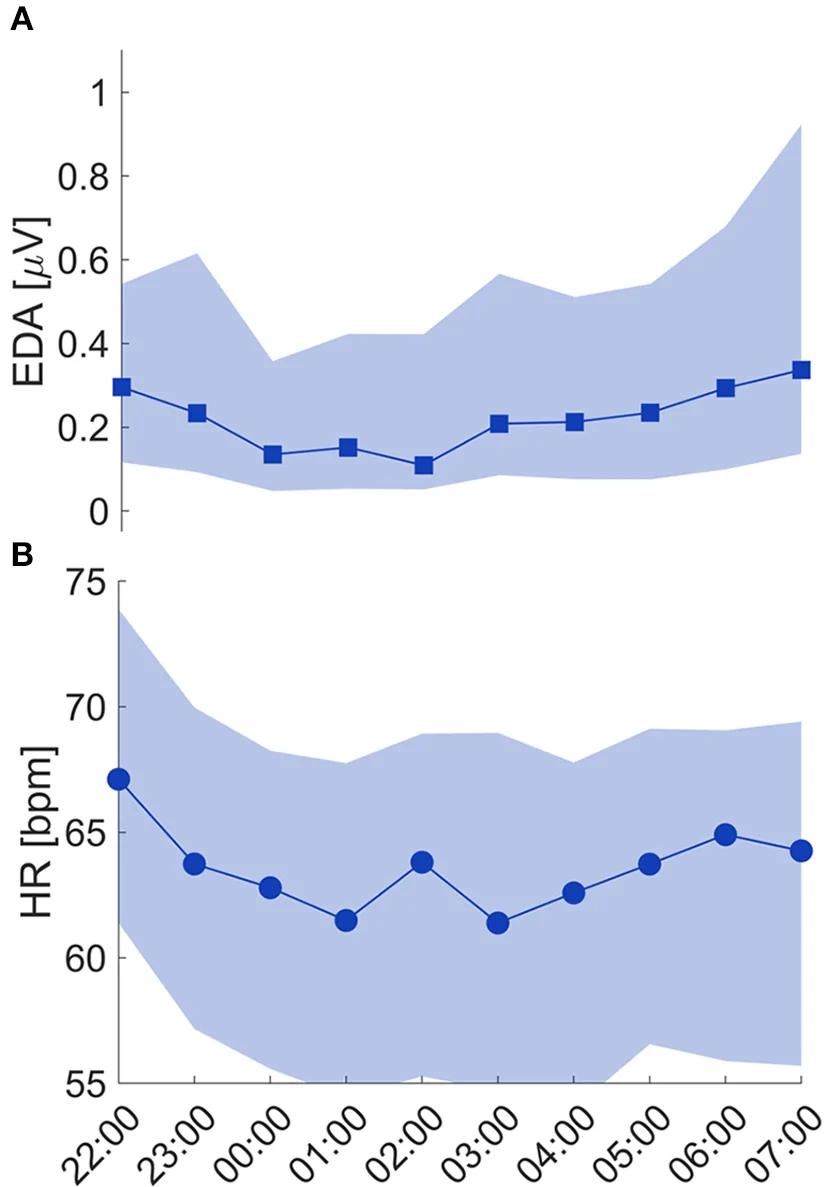
**(A)** Phasic EDA and **(B)** heart rate (HR) during the 10 movies over the course of the night. The markers depict the median across participants, shaded area depict the 25–75th percentile.

To allow comparison of the predictive value of inter-subject correlations with individual physiological responses, we repeated steps three and four of the above analyses with mean EDA or heart rate as predictor. Figure 7 shows the median EDA and heart rate across participants during the movies over the course of the night. Shaded area depicts the 25–75th percentile, to reflect the variation across individuals. Both median EDA and heart rate seem to gradually decrease up to about 02:00, and then steadily increase, except for a relatively high median heart rate at 2:00. Indeed, median EDA and median heart rate are significantly and positively correlated (*r* = 0.18, *p* < 0.001).

Table 5 depicts the HLM statistics corresponding to the analyses with individual mean EDA and heart rate values as predictors. When added as predictor with fixed slope, for EDA (step 3a) the model significantly improves over the model in which EDA was not included as predictor [χ^2^(1, *N* = 438) = 12.52, *p* < 0.001]. For heart rate, the model did not significantly improve [χ^2^ (1, *N* = 438) = 0.88, *p* = 0.347]. EDA has a main significant predictive effect on lapse probability (*p* < 0.01), heart rate has not. For EDA the model did not improve when the predictors were added with random slope to allow variation in the relationship between the predictor and PVT lapse probability across participants, for heart rate the model did improve [χ^2^(1, *N* = 438) = 8.32, *p* = 0.016].

**Table 5:** Statistical parameters of the hierarchical linear models using individuals' average EDA or heart rate (HR) as predictor.

Step	Predictor	−2LL	DF	AIC	χ^2^ −2LL change	*R* ^2^	*t* fixed effect
1		−348.06	2	700.12			
2		−287.44	3	580.87	(1, *N* = 438) = 121.25, *p* < 0.001	0.365	
3a	EDA	−281.18	4	570.35	(1, *N* = 438) = 12.52, *p* < 0.001	0.391	3.58^***^
3b	HR	−287	4	581.99	(1, *N* = 438) = 0.88, *p* = 0.347	0.365	−0.94
4a	EDA	−279.93	6	571.87	(1, *N* = 438) = 2.48, *p* = 0.289	0.410	2.96^**^
4b	HR	−282.84	6	577.67	(1, *N* = 438) = 8.32, *p* = 0.016	0.405	−1.35

^**^p < 0.01. ^***^p < 0.001. −2LL, minus 2 Log Likelihood statistic; DF, degrees of freedom; AIC, Akaike Information Criterion.

When comparing the AIC scores of the predictions using inter-subject correlations and individuals' physiological activation, for heart rate the former appears a better predictor. For EDA, mean EDA has more predictive value than inter-subject correlations in EDA.

### 3.5. Individuals' mean EDA and inter-subject correlations in EDA as predictor of vigilance

As both mean EDA and inter-subject correlations in EDA had a significant main predictive effect on PVT lapse probability, we investigated the potential added value of inter-subject correlations in EDA over one's individual mean phasic EDA. Table 6 shows the statistical parameters of the hierarchical linear model using both individuals' mean EDA and inter-subject correlations in EDA as predictor with random intercept and fixed slope. It shows that that the prediction of lapse probability is significantly better with mean EDA and inter-subject correlations in EDA compared to only mean EDA (*p* = 0.006). As expected, the prediction of lapse probability is also significantly better with mean heart rate and inter-subject correlations in heart rate compared to only mean heart rate (*p* < 0.001).

**Table 6:** Statistical parameters of the hierarchical linear models using individuals' average EDA and inter-subject correlations in EDA (ISC-EDA).

Step	Predictor	−2LL	DF	AIC	χ^2^ −2LL change	*R* ^2^	*t* fixed effect
3c	EDA	−277.49	5	564,98	(1, *N* = 438) = 7.37, *p* = 0.006	0.406	3.72^***^
	ISC-EDA						−2.73^**^
3b	HR	−279.55	5	569.1	(1, *N* = 438) = 14.89, *p* < 0.001	0.388	−1.63
	ISC-HR						−3.89^***^
4c	EDA	−272.52	10	565.04	(1, *N* = 438) = 9.94, *p* = 0.077	0.448	3.44^**^
	ISC-EDA						−2.97^**^
4d	HR	−274.1	10	568.19	(1, *N* = 438) = 10.91, *p* = 0.053	0.442	−1.82
	ISC-HR						−3.22^**^

The Chi-square −2LL change here refers to the change with respect to step 3a of Table 5. ^**^p < 0.01. ^***^p < 0.001. −2LL, minus 2 Log Likelihood statistic; DF, degrees of freedom; AIC, Akaike Information Criterion.

## 4. Discussion

### 4.1. Physiological synchrony as predictor of vigilant attention

The main aim of the current work was to investigate whether variations in vigilant attention that arise from sleep deprivation can be captured by inter-subject correlations in EDA and heart rate as measures of physiological synchrony. To do so we investigated whether inter-subject correlations in the phasic component of EDA and heart rate could predict the variations in subsequent vigilant attentional performance throughout a night of sleep deprivation. We found that inter-subject correlations in EDA had a significant main predictive effect on the performance of a vigilant attention task. This was not the case for inter-subject correlations in heart rate.

Using two separate permutation methods, we established that the inter-subject correlations in EDA and heart rate were higher than expected based on chance for most participants and most movies.

We first replicated the relation between inter-subject correlations in signals recorded during the narrative and narrative retention. Inter-subject correlations in heart rate and EDA were both positively correlated with the number of correctly answered questions about the movie clips. In previous work we only found this relation to be statistically significant for inter-subject correlations in heart rate (Stuldreher et al., 2020b, 2023b). Now we find that in EDA such inter-subject correlations are associated with performance as well. Additionally, we extended previous work by showing that inter-subject correlations do not only capture performance differences between individuals, who received different attentional instructions, or with different personal characteristics, but that inter-subject correlations also capture the variations in performance within an individual. The prediction of the number of correct answers using inter-subject correlations improved when the hierarchical linear model allowed the relation to vary between individuals. This indicates that the relation between inter-subject correlation and attentional performance is different for everyone and confirms the benefit of personalized models in neuroergonomics (Dehais et al., 2020).

Importantly, we also find that the inter-subject correlations in EDA and heart rate during narrative movie clips predict performance on a consecutive vigilant attention task. This suggests that the momentary attentional processing capabilities captured by inter-subject correlations during the presented narrative reflect longer lasting variations in general attentional capability of individuals that also affects other types of tasks. Although significant, the association between inter-subject correlations and vigilant attention, and the predictive value of inter-subject correlations in the model, was modest. This may be explained by a discrepancy in the attentional processes that are captured by inter-subject correlations in response to relatively engaging movies and the subsequent PVT, and the effect sleep deprivation has on each of these processes. Lapses in attention, i.e., short moments of inattentiveness, have been considered the main reason for a decline in cognitive functioning through sleep deprivation (Williams et al., 1959; Kjellberg, 1977), though later it was found that in between lapses cognitive functioning was also impacted through slowing of cognitive processing (Kjellberg, 1977; Dorrian and Dinges, 2005) and fluctuations in alertness (Doran et al., 2001). Still, sleep deprivation especially impacts cognitive functioning in long, simple and monotonous tasks requiring reaction speed or vigilance (Alhola and Polo-Kantola, 2007; Lim and Dinges, 2008; Hudson et al., 2020). The PVT is specifically designed to show a strong effect of sleep deprivation. It is a long monotonous task, that captures the lapses in attention and does not require cognitively complex or emotional processing. Inter-subject correlations, on the other hand, have been reported to capture the cognitive processing of a shared stimulus and to be modulated by the level of “attentional engagement” with the stimulus (Dmochowski et al., 2012; Stuldreher et al., 2020b; Madsen and Parra, 2022). The concept attentional engagement implies a broad type of attention and entails processes like logical reasoning, emotional processing, empathy elicitation and low level visual processing (Dmochowski et al., 2012). The stimuli during which we monitored inter-subject correlations were not simple and monotonous, but relatively engaging movies. Thereby, attending to these movies does not require strong vigilant abilities as the movies' features attract attention automatically in addition to the internal guidance of attention to the movie. Attentional engagement and the inter-subject correlations that are said to capture it, may thus not be influenced as strongly by sleep deprivation as the psychomotor vigilance task. Sleep deprivation generally does not show such a strong decline in performance for tasks that encourage participants to remain engaged and attentive compared to vigilance tasks (Pilcher et al., 2007).

As we presented all participants with the same order of movies, we cannot separate effects of sleep deprivation on inter-subject correlation with the effect of individual movies on inter-subject correlations in the current experiment. In a previous experiment with six of the 10 current movies we did not find the same pattern in the inter-subject correlations in EDA and heart rate as in the current study (Stuldreher et al., 2023b), suggesting it is sleep deprivation that affects the inter-subject correlations over the night and not movie-specific features.

### 4.2. Individuals' physiological activity as predictor of vigilant attention

We compared the predictive value of inter-subject correlations in heart rate and EDA to the predictive value of individual's mean EDA and heart rate. Mean EDA predicted vigilant performance. Inter-subject correlations in heart rate explained more variance of the vigilant attention than individuals' mean heart rate. Inter-subject correlations in EDA explained less variance than individuals' mean EDA. To further investigate the potential added value of inter-subject correlations, we added both individuals' mean physiology and inter-subject correlations in these physiological measures to an HLM. We compared performance to that of the model using only individuals' mean EDA or heart rate. The predictive performance significantly improved when inter-subject correlations were added, indicating that interpersonal analysis of EDA and heart rate is of added value when interested in monitoring attention.

Our finding that EDA was negatively associated with subsequent lapse probability is in line with the established relation between decreased vigilant performance when arousal decreases. Miró et al. (2002) reported a steady decrease in skin conductance throughout the first sleep deprived night, indicating that arousal decreases over the course of a sleep deprived night. Posada-Quintero et al. (2017) found a high negative correlation between the mean values of the skin conductance level and reaction time among sleep deprived individuals, indicating that the arousal decrease indeed results in worse task performance.

## 5. Conclusions

We replicated findings that physiological synchrony in both EDA and HR during presented narratives is associated with performance on questions about the narratives. In addition, we found that physiological synchrony in both EDA and HR was associated with performance on a subsequent vigilant attention task. These findings confirm the association between physiological synchrony and attention. Physiological synchrony captures the attentional processing during the narratives, and proves valuable for capturing more general changes in the attentional state of monitored individuals. Individuals' mean EDA was also associated with performance on the subsequent vigilant attention task but using inter-subject correlations in EDA in addition to mean EDA to yielded better predictions than prediction mean EDA alone. Our results are an important step toward the use of physiological synchrony as implicit measure of shared attention, as we show that variations in the attentional abilities within individuals can be captured. We see physiological synchrony as potential tool to monitor the changes in attentional engagement within and between individuals in a group. It may for instance be used to assist teachers in evaluating the attentional engagement of students in an (online) classroom or to track the shared attention among cooperating teammates.

## Data Availability

The datasets presented in this study can be found in online repositories. The names of the repository/repositories and accession number(s) can be found at: https://osf.io/69u8h/.
