# Correction: Physiological synchrony in heart rate and electrodermal activity predicts decreased vigilant attention induced by sleep deprivation

**DOI:** 10.3389/fnrgo.2026.1965705

**Published:** 2026-10-12

**Authors:** Ivo V. Stuldreher, Emma Maasland, Charelle Bottenheft, Jan B. F. van Erp, Anne-Marie Brouwer

**Affiliations:** 1 Human Performance, Netherlands Organisation for Applied Scientific Research (TNO), Soesterberg, Netherlands; 2 Human Media Interaction, Faculty of Electrical Engineering, Mathematics and Computer Science, University of Twente, Enschede, Netherlands; 3 Human Machine Teaming, Netherlands Organisation for Applied Scientific Research (TNO), Soesterberg, Netherlands; 4 Donders Institute for Brain, Cognition and Behaviour, Radboud University, Nijmegen, Netherlands

**Keywords:** inter-subject correlation, physiological synchrony, electrodermal activity, heart rate, sleep deprivation

Due to an error in the MATLAB code, PVT datapoints that were treated as having been recorded at 6:00 and 7:00 had actually been recorded at 22:00 and 23:00, respectively. All other PVT datapoints had been recorded two hours later than initially reported. This error affects the main results of the paper, as the relationship between electrodermal activity (EDA), heart rate and inter-subject correlations (ISC) in these measures with PVT performance is different than previously reported. In sum, the corrected analyses again show a relationship between ISC and PVT performance, where this relation is clearer than reported before. Now, not only ISC-EDA is a significant predictor of PVT performance, but also ISC-HR. The predictive value of individuals' EDA is also stronger, whereas the predictive value of HR no longer holds. In the following parts, the corrections to each individual part of the paper are described in detail.

In the published article, the **title** was erroneously given as: “Physiological synchrony in electrodermal activity predicts decreased vigilant attention induced by sleep deprivation”. The correct title of the article is “Physiological synchrony in heart rate and electrodermal activity predicts decreased vigilant attention induced by sleep deprivation”.

In the published article, there was an error in Figure 5 as published. Due to an error in the MATLAB code, PVT datapoints that were treated as having been recorded at 6:00 and 7:00 had actually been recorded at 22:00 and 23:00 respectively. All other PVT datapoints should have been shifted to two hours later. The corrected Figure 5 appears below.

**Figure 5:**
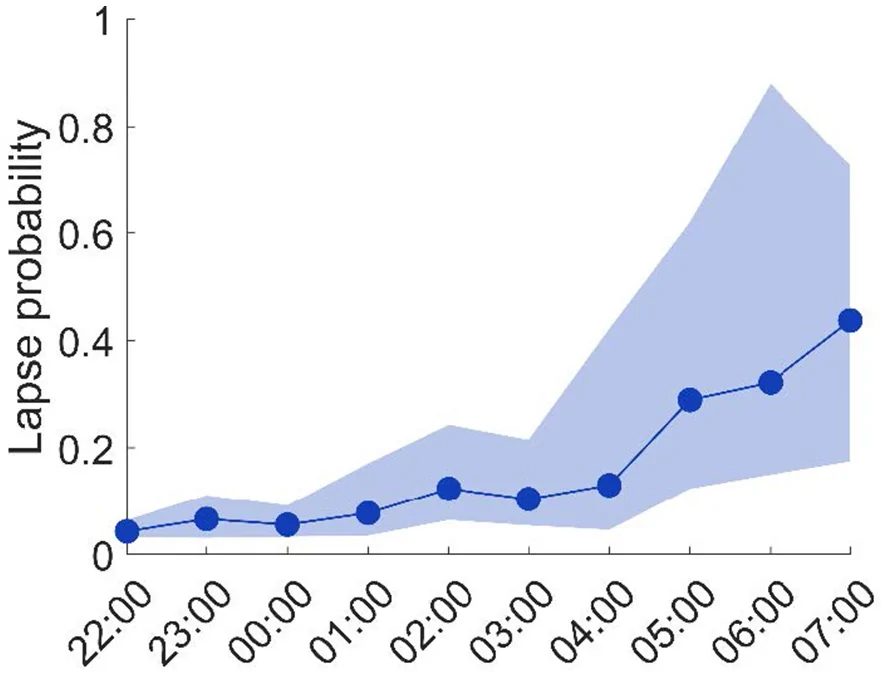
PVT lapse probability over the course of the night. The markers depict the median across participants, shaded area depict the 25–75th percentile.

In the published article, there was an error in Table 4 as published. Due to an error in the MATLAB code, PVT datapoints that were treated as having been recorded at 6:00 and 7:00 had actually been recorded at 22:00 and 23:00 respectively. All other PVT datapoints should have been shifted to two hours later. The consequence of the error was that statistics were erroneously based on shifted data. Correcting this led to the emergence of ISC-HR being a significant predictor of lapse probability, besides only ISC-EDA, which stayed a significant predictor. The corrected Table 4 appears below.

**Table 4:** Statistical parameters of the two hierarchical linear models using inter-subject correlations in either EDA (ISC-EDA) or heart rate (ISC-HR) as predictor of PVT lapse probability.

Step	Predictor	−2LL	DF	AIC	χ^2^ – 2LL change	*R* ^2^	*t* fixed effect
1		−348.06	2	700.12			
2		−287.44	3	580.87	(1, *N* = 438) = 121.25, *p* < 0.001	0.365	–
3a	ISC-EDA	−284.23	4	576.46	(1, *N* = 438) = 6.41, *p* = 0.011	0.379	−2.55^*^
3b	ISC-HR	−280.87	4	569.74	(1, *N* = 438) = 13.14, *p* < 0.001	0.386	−3.65^***^
4a	ISC-EDA	−281.60	6	575.20	(1, *N* = 438) = 5.27, *p* = 0.072	0.396	−2.62^**^
4b	ISC-HR	−280.47	6	572.94	(1, *N* = 438) = 0.80, *p* = 0.670	0.405	−3.42^***^

^*^p < 0.05. ^**^p < 0.01. ^***^p < 0.001. −2LL, minus 2 Log Likelihood statistic; DF, degrees of freedom; AIC, Akaike Information Criterion.

In the published article, there was an error in Table 5 as published. Due to an error in the MATLAB code, PVT datapoints that were treated as having been recorded at 6:00 and 7:00 had actually been recorded at 22:00 and 23:00 respectively. All other PVT datapoints should have been shifted to two hours later. Correcting this led to a stronger predictive value of individuals' average EDA, whereas the predictive value of HR disappeared. The corrected Table 5 appears below.

**Table 5:** Statistical parameters of the hierarchical linear models using individuals' average EDA or heart rate (HR) as predictor.

Step	Predictor	−2LL	DF	AIC	χ^2^ −2LL change	*R* ^2^	*t* fixed effect
1		−348.06	2	700.12			
2		−287.44	3	580.87	(1, *N* = 438) = 121.25, *p* < 0.001	0.365	
3a	EDA	−281.18	4	570.35	(1, *N* = 438) = 12.52, *p* < 0.001	0.391	3.58^***^
3b	HR	−287	4	581.99	(1, *N* = 438) = 0.88, *p* = 0.347	0.365	−0.94
4a	EDA	−279.93	6	571.87	(1, *N* = 438) = 2.48, *p* = 0.289	0.410	2.96^**^
4b	HR	−282.84	6	577.67	(1, *N* = 438) = 8.32, *p* = 0.016	0.405	−1.35

^**^p < 0.01. ^***^p < 0.001. −2LL, minus 2 Log Likelihood statistic; DF, degrees of freedom; AIC, Akaike Information Criterion.

In the published article, there was an error in Table 6 as published. Due to an error in theMATLAB code, PVT datapoints that were treated as having been recorded at 6:00 and 7:00 had actually been recorded at 22:00 and 23:00 respectively. All other PVT datapoints should have been shifted to two hours later. Correcting this led to slight changes for the models that used both average and ISC-EDAmodels. Due to the newly emerged significant predictive value of ISC-HR, we now also created models including average HR and ISC-HR (indicating that ISC-HR added predictive value over HR alone) and added these in the table. The corrected Table 6 appears below.

**Table 6:** Statistical parameters of the hierarchical linear models using individuals' average EDA and inter-subject correlations in EDA (ISC-EDA).

Step	Predictor	−2LL	DF	AIC	χ^2^ −2LL change	*R* ^2^	*t* fixed effect
3c	EDA	−277.49	5	564,98	(1, *N* = 438) = 7.37, *p* = 0.006	0.406	3.72^***^
	ISC-EDA						−2.73^**^
3b	HR	−279.55	5	569.1	(1, *N* = 438) = 14.89, *p* < 0.001	0.388	−1.63
	ISC-HR						−3.89^***^
4c	EDA	−272.52	10	565.04	(1, *N* = 438) = 9.94, *p* = 0.077	0.448	3.44^**^
	ISC-EDA						−2.97^**^
4d	HR	−274.1	10	568.19	(1, *N* = 438) = 10.91, *p* = 0.053	0.442	−1.82
	ISC-HR						−3.22^**^

The Chi-square −2LL change here refers to the change with respect to step 3a of Table 5. ^**^p < 0.01. ^***^p < 0.001. −2LL, minus 2 Log Likelihood statistic; DF, degrees of freedom; AIC, Akaike Information Criterion.

In the published article, there was an error in the **Abstract** as published. Due to an error in the MATLAB code, PVT datapoints that were treated as having been recorded at 6:00 and 7:00 had actually been recorded at 22:00 and 23:00 respectively. All other PVT datapoints should have been shifted to two hours later. In the **Abstract**, we presented the results based on the wrongly shifted PVT datapoints which had led to missing the significant predictive value of ISC-HR for PVT performance. This has been corrected to read:

“**Introduction:** When multiple individuals are presented with narrative movie or audio clips, their electrodermal activity (EDA) and heart rate show significant similarities. Higher levels of such inter-subject physiological synchrony are related with higher levels of attention toward the narrative, as for instance expressed by more correctly answered questions about the narrative. We here investigate whether physiological synchrony in EDA and heart rate during watching of movie clips predicts performance on a subsequent vigilant attention task among participants exposed to a night of total sleep deprivation.

**Methods:** We recorded EDA and heart rate of 54 participants during a night of total sleep deprivation. Every hour from 22:00 to 07:00 participants watched a 10-min movie clip during which we computed inter-subject physiological synchrony. Afterwards, they answered questions about the movie and performed the psychomotor vigilance task (PVT) to capture attentional performance.

**Results:** We replicated findings that inter-subject correlations in EDA and heart rate predicted the number of correct answers on questions about the movie clips. Furthermore, we found that both inter-subject correlations in EDA and heart rate predicted PVT performance. Individuals' mean EDA and heart rate also predicted their PVT performance, but for both EDA and heart rate, combining mean values with inter-subject correlations yielded better explanation of the PVT performance.

**Discussion:** Together, these findings confirm the association between physiological synchrony and attention. Physiological synchrony in EDA and heart rate do not only capture the attentional processing during the time that it is determined, but also proves valuable for capturing more general changes in the attentional state of monitored individuals.”

Due to an error in the MATLAB code, PVT datapoints that were treated as having been recorded at 6:00 and 7:00 had actually been recorded at 22:00 and 23:00 respectively. All other PVT datapoints should have been shifted to two hours later. Correcting this led to the emergence of ISC-HR being a significant predictor of lapse probability, besides only ISC-EDA (which stayed a significant predictor). It also led to a stronger predictive value of individuals' average EDA, whereas the predictive value of HR disappeared, when predicting lapse probability. Due to the newly emerged significant predictive value of ISC-HR, we also created models including average HR and ISC-HR, indicating that ISC-HR added predictive value over HR alone. These updated results demand an updated discussion, in which we present the corrected statistics.

A correction has been made to **Section 3**. The following sections have been adapted to state the following:

“*3.3 Inter-subject correlations in EDA or heart rate as predictor of vigilance*

Next, we investigated the potential association between inter-subject correlation in EDA and heart rate and performance on a subsequent attentional task, i.e., the PVT. A premise for our analysis is that vigilant performance is affected by sleep deprivation and thus varies over the course of the night. Figure 5 shows the PVT lapse probability over the course of the night. The median lapse probability gradually increases from close to zero up to 0.4 at 07:00 o'clock. Consistent with previous findings are the large individual differences in vigilant performance during the night (Hudson et al., 2020), indicated by the shaded area in the figure.

A second premise for inter-subject correlations to be predictive of vigilant performance throughout the night is that the intersubject correlations vary throughout the night. Figure 6 shows inter-subject correlations in EDA and heart rate over the course of the night. Inter-subject correlations in heart rate do not seem to follow a consistent pattern throughout the night. Inter-subject correlations in EDA seem more consistent with the overall lapse probability. Except for the last datapoint at 7:00, the figure suggests a decreasing trend of inter-subject correlations in EDA.

Table 4 summarizes the results of the four steps of HLM analyses used to investigate how well inter-subject correlations in either EDA or heart rate predict PVT lapse probability. In step one, only the dependent variable PVT lapse probability was added to the model to serve as a baseline. The AIC in this model is 700.12.

In step two, the level two variable participant was added to the model to investigate whether allowing for individual differences improves the model prediction. The AIC in this model is 580.87. There is a highly significant change in the −2LL, χ^2^(1, *N* = 438) = 121.21, *p* < 0.001. This step of the model explains 36.5% of the variance in PVT lapse probability. The fixed effect of individual on PVT lapse probability is highly significant (*p* < 0.001).

In step three, the predictor, being inter-subject correlations in either EDA (step 3a) or heart rate (step 3b), was added to the model with a fixed slope and random intercept. The results in this step indicate whether the predictor has a main predictive effect on PVT lapse probability. Adding the predictor with a fixed slope means that the model assumes that for all individuals, the relationship between the predictor and PVT lapse probability is identical (Grinsted et al., 2004). In this step, the AIC value is 576.46 for EDA and 569.74 for heart rate. The −2LL value for EDA is −284.23, a significant improvement compared to the previous step, χ^2^(1, *N* = 438) = 6.41, *p* = 0.011. The total variance explained is 37.9% and the fixed effect is significant (*p* < 0.05). For heart rate, the −2LL value (−280.87) is also significantly lower than the model in step two, χ^2^(1, *N* = 438) = 13.14, *p* < 0.001. The total variance explained is 38.6% and fixed effect is highly significant (*p* < 0.001).

In step four, the predictor variable was added with a random slope and a random intercept, meaning that the model allows the relationship between inter-subject correlations and PVT lapse probabilities to vary between individuals. Neither for EDA (step 4a) nor for heart rate (step 4b) the model in this step was significantly better than the model in step three (EDA: AIC = 575.20, −2LL = −281.60, −2LL χ^2^ (2, *N* = 438) = 05.27, *p* = 0.072; heart rate: AIC = 572.94, −2LL = −280.47, −2LL χ^2^ (2, *N* = 438) = 0.80, *p* = 0.670).

*3.4 Individuals' mean EDA or heart rate as predictor of vigilance*

To allow comparison of the predictive value of inter-subject correlations with individual physiological responses, we repeated steps three and four of the above analyses with mean EDA or heart rate as predictor. Figure 7 shows the median EDA and heart rate across participants during the movies over the course of the night. Shaded area depicts the 25–75th percentile, to reflect the variation across individuals. Both median EDA and heart rate seem to gradually decrease up to about 02:00, and then steadily increase, except for a relatively high median heart rate at 2:00. Indeed, median EDA and median heart rate are significantly and positively correlated (r = 0.18, p < 0.001).

Table 5 depicts the HLM statistics corresponding to the analyses with individual mean EDA and heart rate values as predictors. When added as predictor with fixed slope, for EDA (step 3a) the model significantly improves over the model in which EDA was not included as predictor [χ^2^(1, *N* = 438) = 12.52, *p* < 0.001]. For heart rate, the model did not significantly improve [χ^2^ (1, *N* = 438) = 0.88, *p* = 0.347]. EDA has a main significant predictive effect on lapse probability (*p* < 0.01), heart rate has not. For EDA the model did not improve when the predictors were added with random slope to allow variation in the relationship between the predictor and PVT lapse probability across participants, for heart rate the model did improve [χ^2^(1, *N* = 438) = 8.32, *p* = 0.016].

When comparing the AIC scores of the predictions using inter-subject correlations and individuals' physiological activation, for heart rate the former appears a better predictor. For EDA, mean EDA has more predictive value than inter-subject correlations in EDA.

*3.5 Individuals' mean physiology and inter-subject correlations as predictor of vigilance*

As both mean EDA and inter-subject correlations in EDA had a significant main predictive effect on PVT lapse probability, we investigated the potential added value of inter-subject correlations in EDA over one's individual mean phasic EDA. Table 6 shows the statistical parameters of the hierarchical linear model using both individuals' mean EDA and inter-subject correlations in EDA as predictor with random intercept and fixed slope. It shows that that the prediction of lapse probability is significantly better with mean EDA and inter-subject correlations in EDA compared to only mean EDA (*p* = 0.006). As expected, the prediction of lapse probability is also significantly better with mean heart rate and inter-subject correlations in heart rate compared to only mean heart rate (*p* < 0.001).”

A correction has been made in the **Section 4**. In *Section 4.1*, paragraph 4, the sentence “Importantly, we also find that the inter-subject correlations in EDA during narrative movie clips predict performance on a consecutive vigilant attention task.” was corrected to “Importantly, we also find that the inter-subject correlations in EDA and heart rate during narrative movie clips predict performance on a consecutive vigilant attention task.”

A correction has been made in the **Section 4**. In *Section 4.2*, paragraphs 1 and 2 were corrected from:

“We compared the predictive value of inter-subject correlations in heart rate and EDA to the predictive value of individual's mean EDA and heart rate. Mean EDA and heart rate both predicted vigilant performance. Since inter-subject correlations in heart rate did not predict performance at all, the mean heart rate thus had a higher predictive value. The mean EDA explained less variance of the vigilant attention than inter-subject correlations in EDA. Also when comparing the AIC, inter-subject correlations in EDA seemed to provide a better model fit than the mean value. To further investigate the potential added value of inter-subject correlations, we added both individuals' mean EDA and inter-subject correlations in EDA to an HLM. We compared performance to that of the model using only individuals' mean EDA. The predictive performance was found to significantly improve when inter-subject correlations were added, indicating that interpersonal analysis of EDA is of added value when interested in monitoring attention.

Our finding that EDA and heart rate were negatively associated with subsequent lapse probability is in line with the established relation between decreased vigilant performance when arousal decreases. Miró et al. (2002) reported a steady decrease in skin conductance throughout the first sleep deprived night, indicating that arousal decreases over the course of a sleep deprived night. Posada-Quintero et al. (2017) found a high negative correlation between the mean values of the skin conductance level and reaction time among sleep deprived individuals, indicating that the arousal decrease indeed results in worse task performance. Van Den Berg and Neely (2006) found similar effects using heart rate, as they reported a strong negative correlation between heart rate and reaction time in sleep deprived individuals.” to:

“We compared the predictive value of inter-subject correlations in heart rate and EDA to the predictive value of individual's mean EDA and heart rate. Mean EDA predicted vigilant performance. Inter-subject correlations in heart rate explained more variance of the vigilant attention than individuals' mean heart rate. Inter-subject correlations in EDA explained less variance than individuals' mean EDA. To further investigate the potential added value of inter-subject correlations, we added both individuals' mean physiology and inter-subject correlations in these physiological measures to an HLM. We compared performance to that of the model using only individuals' mean EDA or heart rate. The predictive performance significantly improved when inter-subject correlations were added, indicating that interpersonal analysis of EDA and heart rate is of added value when interested in monitoring attention.

Our finding that EDA was negatively associated with subsequent lapse probability is in line with the established relation between decreased vigilant performance when arousal decreases. Miró et al. (2002) reported a steady decrease in skin conductance throughout the first sleep deprived night, indicating that arousal decreases over the course of a sleep deprived night. Posada-Quintero et al. (2017) found a high negative correlation between the mean values of the skin conductance level and reaction time among sleep deprived individuals, indicating that the arousal decrease indeed results in worse task performance.”

A correction has been made in **Section 5**. This paragraph has been corrected from: “We replicated findings that physiological synchrony during presented narratives is associated with performance on questions about the narratives. In addition, we found that physiological synchrony was associated with performance on a subsequent vigilant attention task. These findings confirm the association between physiological synchrony and attention. Physiological synchrony captures the attentional processing during the narratives, and proves valuable for capturing more general changes in the attentional state of monitored individuals. The discrepancy in findings using EDA and heart rate suggest that physiological synchrony in these measures captures different aspects of attentional processing. Physiological synchrony in EDA may especially reflect short moments of attentiveness caused by arousing sensory events. Synchrony in heart rate may rather reflect longer intervals of attention driven by higher-level cognitive control. Individuals' mean EDA and heart rate were also associated with performance on the subsequent vigilant attention task. For EDA, inter-subject correlations explained more variance if vigilant performance than individual's mean activity. Using inter-subject correlations in EDA in addition to mean EDA to predict vigilant performance yielded better performance than prediction based on mean EDA alone. Our results are an important step toward the use of physiological synchrony as implicit measure of shared attention, as we show that variations in the attentional abilities within individuals can be captured. We see physiological synchrony as potential tool to monitor the changes in attentional engagement within and between individuals in a group. It may for instance be used to assist teachers in evaluating the attentional engagement of students in an (online) classroom or to track the shared attention among cooperating teammates.” to:

“We replicated findings that physiological synchrony in both EDA and HR during presented narratives is associated with performance on questions about the narratives. In addition, we found that physiological synchrony in both EDA and HR was associated with performance on a subsequent vigilant attention task. These findings confirm the association between physiological synchrony and attention. Physiological synchrony captures the attentional processing during the narratives, and proves valuable for capturing more general changes in the attentional state of monitored individuals. Individuals' mean EDA was also associated with performance on the subsequent vigilant attention task but using inter-subject correlations in EDA in addition to mean EDA to yielded better predictions than prediction mean EDA alone. Our results are an important step toward the use of physiological synchrony as implicit measure of shared attention, as we show that variations in the attentional abilities within individuals can be captured. We see physiological synchrony as potential tool to monitor the changes in attentional engagement within and between individuals in a group. It may for instance be used to assist teachers in evaluating the attentional engagement of students in an (online) classroom or to track the shared attention among cooperating teammates.”

The *Section 4.3* has been removed from the article.

The original version of this article has been updated.

