# Supplementary material for: Physiological synchrony in electrodermal activity predicts decreased vigilant attention induced by sleep deprivation
Source: Front Neuroergon. 2023 Jun 29;4:1199347. doi: 10.3389/fnrgo.2023.1199347 (PMC10790929; doi:10.3389/fnrgo.2023.1199347)
Supplement: Supplementary file 1 [file Data_Sheet_1.docx]

Supplementary Material

Physiological synchrony in electrodermal activity predicts decreased vigilant attention induced by sleep deprivation

Ivo V. Stuldreher*, Emma Maasland, Charelle Bottenheft, Jan B. F. van Erp, Anne-Marie Brouwer

*** Correspondence:** Ivo Stuldreher: ivo.stuldreher@tno.nl

Supplementary Table 1. Questions and answer options belonging to the 10 movie clips, all in Dutch. The leftmost answer is the correct answer. In the experiment the answers were presented in random order.

| Movie 1: Chauffeur | | | |
| --- | --- | --- | --- |
| Wat is de kleur van de dienstauto van de chauffeur? | | | |
| Zwart | Blauw | Groen | Grijs |
| Wat vraagt de chauffeur aan de twee kinderen die vlak bij zijn auto staan op het schoolplein? | | | |
| Of ze die jongen op het schoolplein kennen | Of ze weten wat het adres hier is | Of ze met hem mee willen gaan naar in ruil voor een cadeau | Of ze hem kunnen vertellen hoe laat het is |
| Hoe heet de jongen die met de chauffeur mee gaat? | | | |
| Tom | Tim | Pim | Sam |
| Wat belooft de chauffeur de jongen als hij mee gaat? | | | |
| Een speelgoed auto | Een milkshake | Dat hij een stukje mag rijden | Dat hij hem naar huis brengt |
| Wat doen de chauffeur en de jongen in de auto op het industrie terrein? | | | |
| De jongen mag een stukje rijden in de auto | Ze gaan op bezoek bij een van de klanten van de chauffeur | Ze praten over de band die ze hebben | Ze eten fastfood |
| Wat doen de chauffeur en de jongen direct nadat de jongen zelf een stukje heeft gereden? | | | |
| Ze gaan langs een fastfood restaurant | Ze gaan door de autowasstraat | Ze rijden terug naar de school van de jongen | Ze rijden terug naar het huis van de jongen |
| Wat bestelt de jongen bij het restaurant? | | | |
| Een hamburger en een vanillemilkshake | Kipnuggets en een vanillemilkshake | Een hamburger en een aardbeienmilkshake | Kipnuggets en een aardbeienmilkshake |
| Wat is de relatie tussen de jongen en de chauffeur? | | | |
| Vader - Zoon | Chauffeur - Klant | Oom - Neefje | De jongen kent de chauffeur niet |
| Waarom wordt de chauffeur boos en zegt hij ‘godverdomme’ tegen de jongen in de auto? | | | |
| De jongen morst zijn drinken in de auto | De jongen rijdt met de auto ergens tegenaan | De jongen wil maar niet luisteren naar de chauffeur | De jongen wil vluchten van de chauffeur |
| Wat zegt de chauffeur tegen de moeder van de jongen? | | | |
| De chauffeur zegt niets | Lul | Klootzak | Hoi |

| Movie 2: El Mourrabi | | | |
| --- | --- | --- | --- |
| Wat is de naam van deze film? | | | |
| El Mourabbi | Galeria | El Galeria | Lost and found |
| Hoe reageert de pizzabezorger op zijn fooi? | | | |
| Boos, hij scheldt de gever uit | Blij, hij bedankt de gever | Teleurgesteld, maar hij zegt de gever niks | Routinematig, je merkt niks bijzonders aan hem |
| Hoe heet de pizzabezorger? | | | |
| Rachid | Willem | Mourabbi | Achmed |
| Op wat voor kleur scooter rijdt de pizzabezorger? | | | |
| Rood | Zwart | Wit | Blauw |
| Welke kleur kleren heeft de baby? | | | |
| Grijs / blauw | Zwart / wit | Grijs / zwart | Wit / blauw |
| Waar verstopt Rachid de baby voor zijn vrienden en collega's buiten het pizzarestaurant? | | | |
| In de bak achterop zijn scooter | Onder het zadel van zijn scooter | In een portiek | Achter een geparkeerde auto |
| Waarom zet de pizzabezorger de muziek op zijn kamer ineens zo hard? | | | |
| Om het geluid van de huilende baby te maskeren | Om zijn frustratie te uiten | Omdat hij boos is op zijn zus en haar dwars wil zitten | Om de baby wakker te maken |
| Hoe heet het pizzarestaurant waar de bezorger voor werkt? | | | |
| Galeria | Mourrabi | Dominos | Il Duomo |
| Waar geeft de pizzabezorger de baby af? | | | |
| Bij het ziekenhuis | Bij zijn eigen ouders | Bij de ouders van de baby | Bij het station |
| Welke dieren bekeken de pizzabezorger en baby? | | | |
| Geiten | Kippen | Koeien | Ezels |

| Movie 3: De Chinese Muur | | | |
| --- | --- | --- | --- |
| Wat bestelt de vrouwelijke hoofdpersoon in het begin van de film? | | | |
| Tomatensoep met extra veel kip | Kippensoep met extra veel kip | Tomatensoep met extra gehaktballetjes | Groentesoep met extra gehaktballetjes |
| Aan wie doet de man, die aan een van de andere tafels zit, de vrouwelijke hoofdpersoon aan denken? | | | |
| Fred | Roberts | Kees | Bert |
| In welke ijs heeft de vrouwelijke hoofdpersoon zin? | | | |
| Dame Blanche | Bananensplit | Aardbeiencoupe | Vanillecoupe |
| Waarom zingt iedereen in het restaurant voor de vrouwelijke hoofdpersoon? | | | |
| Omdat ze zei dat ze jarig was, terwijl dat niet het geval is | Omdat de restaurantbezoekers hoorden dat ze jarig is | De restaurantbezoekers zijn in het restaurant om de vrouw te verassen | De restaurantbezoekers zingen helemaal niet voor de vrouwelijke hoofdpersoon |
| Waarom is het niet gek dat de personen tegenover de vrouwelijke hoofdpersoon op elkaar lijken? | | | |
| Ze zijn broer en zus | Ze zijn al zo lang bij elkaar | Ze zijn (niet verder gespecificeerde) familie | Ze zijn samen een tweeling |
| Hoe komen de gasten in het restaurant over op de persoon die op het einde binnenkomt om Chinees af te halen? | | | |
| Als een grote happy family | Als een partij feestgangers | Als burgerlijke types | Als vaste gasten van het restaurant |
| Hoeveel sterretjes stonden er op de taart? | | | |
| Vier | Twee | Drie | Tien |
| Wat was er aan de hand met de vrouw van Fred? | | | |
| Ze was dement | Ze had kanker | Ze was depressief | Ze had een spierziekte |
| Hoe heet de zoon van de vrouwelijke hoofdpersoon? | | | |
| Robert | Fred | Peter | Bert |
| Hoe dacht de vrouwelijke hoofdpersoon dat de groep jonge mensen elkaar zou kennen, en hoe kenden ze elkaar daadwerkelijk? | | | |
| Collega's in de IT sector / lid van dezelfde schaatsclub | Collega's bij een accountantkantoor / lid van dezelfde hockeyclub | Lid van dezelfde schaatsclub / collega's in de IT sector | Lid van dezelfde hockeyclub / collega's bij een accountantkantoor |

| Movie 4: One of the Boys | | | |
| --- | --- | --- | --- |
| Wat voor fruit ligt er in de kleedkamer? | | | |
| Banaan | Appel | Kiwi | Mandarijn |
| Waar is de hoofdpersoon op vakantie geweest? | | | |
| Drenthe | Mallorca | Ibiza | Texel |
| Wat is volgens de hoofdrolspeler een wet van de natuur? | | | |
| Tijdens de zomervakantie worden alle chicks lekkerder | Tijdens de zomervakantie worden alle chicks hitsiger | Tijdens de zomervakantie vinden alle jongens meisjes lekkerder | Tijdens de zomervakantie worden alle jongens hitsiger |
| Hoe heet de hoofdrolspeler? | | | |
| Daan | Dan | Bas | Bastiaan |
| Waarom wordt de hoofdrolspeler boos en agressief? | | | |
| Omdat zijn mannelijke teamgenoten Britt lastig vallen | Omdat het sporten hem niet zo goed afgaat | Omdat Britt onaardig tegen hem doet | Omdat hij wordt lastig gevallen door een aantal van zijn teamgenoten |
| Wat doet de hoofdrolspeler wanneer hij samen met het meisje in de kleedkamer is? | | | |
| Hij geeft haar een kus | Hij troost haar en laat haar weer gaan | Hij ruziet met haar | Hij negeert haar en pakt zijn spullen |
| Welke sport speelt het team in deze film? | | | |
| Korfbal | Handbal | Basketbal | Trefbal |
| Wat staat er op het briefje dat aan de muur hangt in de kleedkamer? | | | |
| Is het ballenhok dicht? | Staan de lichten uit? | Is de kantine op slot? | Is het hek op slot? |
| Welke kleur heeft de bal? | | | |
| Geel / Blauw | Geel / Zwart | Wit / Blauw | Wit / Zwart |
| Welke kleur is het shirt dat de gezette jongen draagt? | | | |
| Roze | Rood | Geel | Wit |

| Movie 5: Samual | | | |
| --- | --- | --- | --- |
| Wat is de naam van deze film? | | | |
| Samual | Amsterdam | Owusu | On the other side |
| Hoe komt Samual in eerste instantie aan geld? | | | |
| Bedelen op straat | Werken in de haven | Meegenomen op reis | Lenen bij een geldkantoor |
| Wat doet Samual met het muntgeld dat hij heeft? | | | |
| Hij probeert te bellen bij een telefooncel | Hij koopt er koffie van | Hij betaalt zijn vervoer ermee | Hij stopt het in zijn zak voor later |
| Waarom is Samual in Amsterdam? | | | |
| Om zijn dochter te bezoeken | Omdat hij op de vlucht is geslagen | Om werk te zoeken | Hij zoekt zijn geboorteplaats weer op |
| Waar zijn volgens Samual de dromen van de toekomst? | | | |
| Aan de overkant van de zee | Geschreven in de wolken | Diep in je hart | Overal om je heen |
| Aan wie wilt Samual een briefje geven bij vertrek uit het politiebureau? | | | |
| Aan het meisje in het kinderdagverblijf | Aan de begeleider van het kinderdagverblijf | Aan een van de begeleidende agenten | Aan zijn dochter |
| Aan wie doet het meisje in het kinderdagverblijf Samual denken? | | | |
| Aan zijn eigen dochter | Aan de meisjes in zijn dorp | Aan een meisje zonder zorgen | Aan een typisch westers opgevoed kind |
| Hoe maakt de geldiwsselaar duidelijk dat hij geen geld kan wisselen? | | | |
| Door zijn hoofd te schudden | Door te zeggen dat hij geen geld kan wisselen | Door afwijzend te kijken | Door Samual te negeren |
| Wat staat er op het briefje dat Samual bij zich had? | | | |
| Een telefoonnummer | Een adres | Een naam | Een instantie |
| Wat staat er op het raam van het kinderdagverblijf geschilderd? | | | |
| Een konijn | De maan | Een beer | Vlaggetjes |

| Movie 6: Turn it Around | | | |
| --- | --- | --- | --- |
| Wat is de naam van deze film? | | | |
| Turn it around | One of the boys | Outcoming | One of the friends |
| Wie is 'het lekkerste wijf op het feest'? | | | |
| Denise | Isabel | Karen | Floor |
| Aan wie wordt hoofdpersoon Bram op zijn verzoek voorgesteld? | | | |
| Florian | Denise | Isabel | Bas |
| Waar komt Florian vandaan? | | | |
| Amsterdam | Het Gooi | Rotterdam | Twente |
| Wat is de reden dat Bram en Florian naar buiten gaan? | | | |
| Binnen is het te druk | Binnen is het te warm | Buiten is het gezelliger | Buiten staat het drinken |
| Bij welke term schrikt Bram terwijl zijn hand dichter naar die van Florian kruipt? | | | |
| Vuile Flikker! | Homo! | Eikel! | Nicht! |
| Welk spel speelt de groep in deze film? | | | |
| Flesje draaien | Doen, durf of de waarheid | Het kusspel | Het spel heeft geen naam |
| Waarom wilt Bram tegen het einde van de film nog Flesje draaien? | | | |
| Omdat Florian naar huis gaat | Omdat hij met Denise wilt zoenen | Omdat hij al een tijdje wacht op zijn beurt | Omdat hij de druk voelt van de groep om mee te doen. |
| Komt het Flesje van Bram uit bij Floor? | | | |
| Nee, maar toch zoent hij hem | Ja, daarom zoent hij hem | Nee, daarom zoent hij hem niet | Ja, maar toch zoent hij hem niet. |
| Wat is de kleur van het etiket op het flesje? | | | |
| Blauw | Wit | Groen | Zwart |

| Movie 7: En Route | | | |
| --- | --- | --- | --- |
| Wat drinken de gezinsleden bij het ontbijt? | | | |
| Melk | Water | Jus d'Orange | Niets |
| Welk huisdier heeft het gezin naast de wandelende tak? | | | |
| Geen ander huisdier | Een vogel | Een hamster | Een poes |
| Waarom maken Inay en haar broertje geen haast? | | | |
| Uit ervaring weten ze dat ze dan beloond worden me toetjes | Ze willen meer tijd om lekker te spelen | Ze zijn boos op papa en willen hem terugpakken door hem te laat te laten komen | Ze hopen dat ze dan de volgende keer thuis mogen blijven. |
| Wat is de kleur van Inays jas? | | | |
| Roze | Zilverkleurig | Lichtblauw | Groen |
| Wat is er afgebeeld op de tekening die te zien is aan het begin van de film? | | | |
| Een huis | Een poes | Een regenboog | Een gezin |
| Wat moeten ze nog uit de keuken halen? | | | |
| De tassen | De portemonnee | Een sjaal | De sleutels |
| Hoeveel mensen vraagt vader om voedsel? | | | |
| Vier | Twee | Drie | Een |
| Wie zeggen samen hetzelfde getal waardoor de drie af zijn bij het telspel? | | | |
| Inay en haar broertje | Inay en haar vader | Inay's broertje en haar vader | Alle drie |
| Welke vogels eten het brood op straat? | | | |
| Meeuwen | Eenden | Mussen | Duiven |
| Wat krijgt de vader van Inay van de eerste man die hij aanspreekt bij de voedselbank? | | | |
| Een prei | Een wortel | Een brood | Meer toetjes |

| Movie 8: Mowgli en Fidel | | | |
| --- | --- | --- | --- |
| Wat ligt of staat er niet op de rand van het bad? | | | |
| Een spons | Een glas | Een blikje | Een asbak |
| Wat moet Fidel volgens Mowgli doen tegen zijn denkstoornis? | | | |
| Een jointje draaien | Wat eerder gaan slapen | Een borrel drinken | Stoppen met piekeren |
| Wat moet er volgens Fidel nog gebeuren voor de twee weg kunnen gaan? | | | |
| De koelkast moet nog uitgeruimd worden | De planten moeten nog water | Het water moet nog afgesloten worden | Het gas moet nog afgesloten worden |
| Welke kleur is de auto van de chauffeur? | | | |
| Rood | Zwart | Blauw | Geel |
| Wat vindt Fidel een fijn idee? | | | |
| Dat er tussen Bangkok en Amsterdam geen zee zit | Dat je vanaf Bangkok binnen een dag weer in Amsterdam kan zijn | Dat er veel schepen varen tussen Bangkok en Rotterdam | Dat het een directe vlucht is |
| Wat voor jas draagt Fidel? | | | |
| Een blauw spijkerjack? | Een blauwe winterjas | Een groen leren jack | Een groene zomerjas |
| Hoe heet de kat? | | | |
| Poetsie | Mimi | Skattie | Kats |
| Welke sticker staat op de brievenbus van he thuis van Mowgli en Fidel? | | | |
| Nee - Nee | Ja - Nee | Ja - Ja | Er staat geen sticker op |
| Wat voor bagage heeft Mowgli bij zich? | | | |
| Een rugtas en weekendtas | Een koffer en een rugtas | Een grote en kleine koffer | Een weekendtas en een koffer |
| Hoe ziet de schoonmaakster in de toiletruimte eruit? | | | |
| Lang bruin haar in een staart | Donkere huidskleur | Blond haar | Felle lippenstift |

| Movie 9: Heen en Weer Dag | | | |
| --- | --- | --- | --- |
| Welke kleur zijn de strepen van het t-shirt van Linus? | | | |
| Blauw met wit | Zwart met wit | Blauw met rood | Rood met wit |
| Wat voor spelletje speelt Linus met de hond? | | | |
| Hij schiet met het waterpistool in het gras | Hij schiet met het waterpistool op de grond | Hij schiet met het waterpistool op zijn vader | Hij schiet met het waterpistool op de buurman |
| Wat draagt de vader van Linus voor kleding? | | | |
| Een witte polo en een bruine korte broek | Een blauwe polo en een bruine korte broek | Een witte polo en een zwarte korte broek | Een blauwe polo en een zwarte korte broek |
| Wat staat of ligt er in het gras als vader de krant leest? | | | |
| Een deel van de krant | Een bal | Een waterpistool | Een blikje bier |
| Wat zit er op de pizza van Linus’ vader? | | | |
| Tonijn | Ansjovis | Mozzarella | Kip |
| Wat zegt de vader van Linus wanneer Linus vraagt: “Moeten we helpen? | | | |
| Ik doe niks | Ik hoef niks te doen | Mij niet gezien | Ik ga niet helpen |
| Linus kijkt in een doos met oude spullen. Wat houdt hij in zijn handen? | | | |
| Een foto en de afdruk van een baby voetje | Een foto en een oud knuffeltje | Een oud knuffeltje en de afdruk van een baby voetje | Een oud knuffeltje en een speen |
| Wat zegt Linus tegen de nieuwe vriend van zijn moeder? | | | |
| Je hebt gemorst | Je hebt een vlek | Je shirt is vies | Je hebt wat laten vallen |
| Waarop zit Linus tijdens het eten van de pizza? | | | |
| Op een vuilnisbak | Op een stoel | Op een kruk | Op een stapel dozen |
| Welke kleur is het huis van Linus? | | | |
| Beige | Blauw | Bruin | Roze |
| Lang bruin haar in een staart | Donkere huidskleur | Blond haar | Felle lippenstift |

| Movie 10: Gutmensch | | | |
| --- | --- | --- | --- |
| Wat is de naam van de baby van Marjolijn? | | | |
| Otto | Theo | Otmar | Oscar |
| Over welk land gaat het als Marjolijn vraagt: “ heb je iets met…? | | | |
| China | Japan | Thailand | India |
| Wat moet Zohre volgens Marjolijn doen met de plant? | | | |
| Nieuwe aarde geven | Goed water geven | Af en toe sproeien | Af en toe plantvoeding geven |
| Wat zegt Zohre dat ze wil als Marjolijn het haar vraagt? | | | |
| Naar een film | Een gewoon leven | Haar familie weer zien | Haar rijbewijs |
| Over welke serie vertelt Marjolijn? | | | |
| GTST | Gooische vrouwen | Voetbalvrouwen | Onderweg naar Morgen |
| Wat vergeet Zohre bijna na de eerste keer bij Marjolijn? | | | |
| Haar sjaal | Haar handschoenen | Haar tas | Haar telefoon |
| Wat is de nieuwe naam die Zohre voor zichzelf kiest op de school? | | | |
| Narcis | Roos | Hyacinth | Hortensia |
| Waarmee wil Marjolijn door Wilma haar kan doorverbonden worden? | | | |
| De afdeling leven | De belastingdienst | De manager | Wilma's baas |
| Om wie maakt Zohre zich zorgen? | | | |
| Haar broertje | Haar tante | Haar vader | Haar moeder |
| Wat voor jas heeft Marjolijn aan in de auto? | | | |
| Een blauw spijkerjack | Een gele regenjas | Een groen jack | Een blauwe regenjas |

**
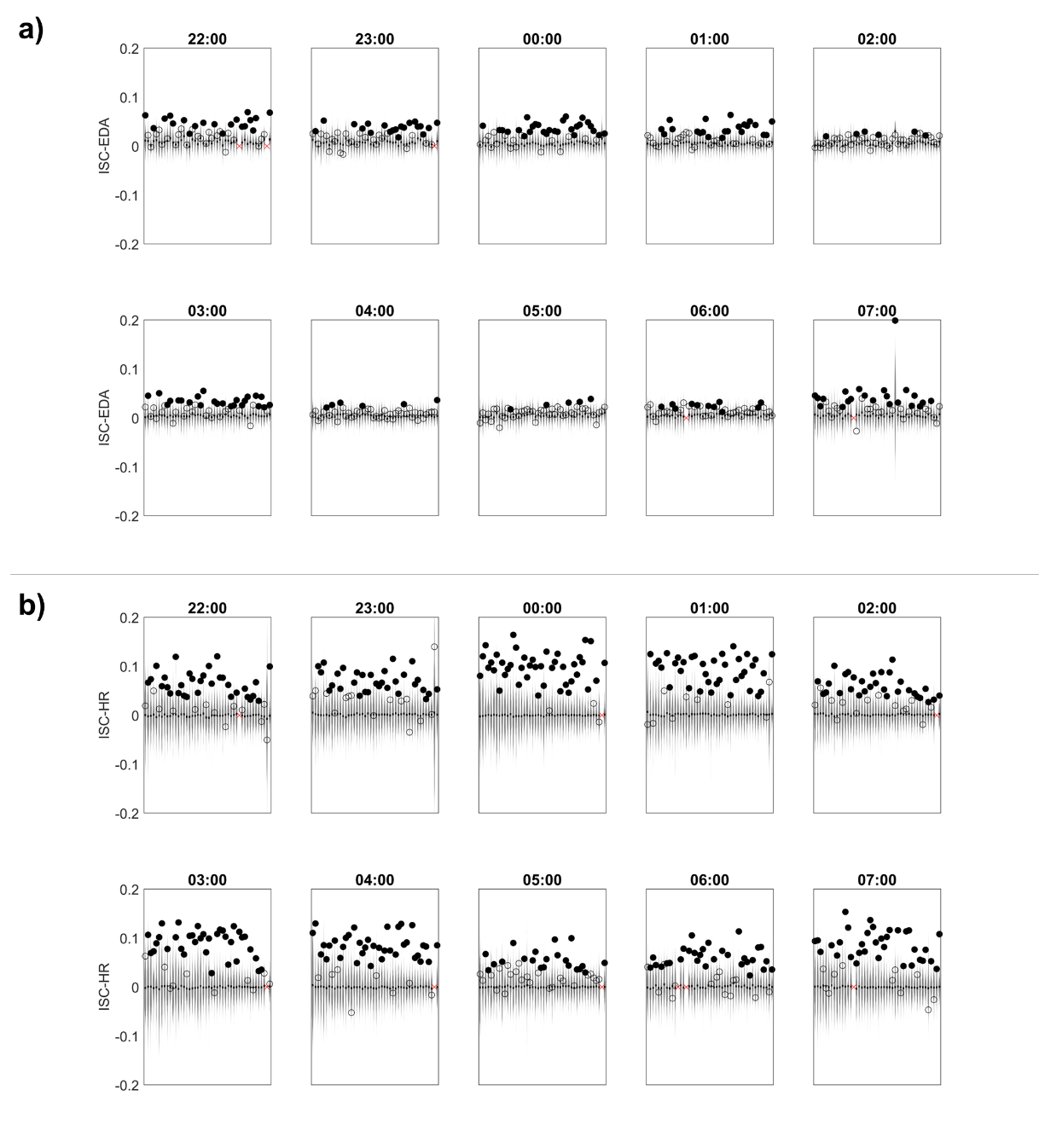
**

**Supplementary Figure 1.** Inter-subject correlations in EDA (ISC-EDA) and heart rate (ISC-HR) for each individual and each movie clip compared to a chance level distribution obtained through circular shuffling. Each marker refers to the participant-to-group inter-subject correlations of a participant. Filled markers depict inter-subject correlations significantly higher than chance, open markers depict inter-subject correlations not higher than chance level. To allow comparison of ISC scores of individuals, missing data are depicted with red crosses.

Supplementary Table 2. t-test statistics comparing inter-subject correlations when videos are randomly swapped between participants with the original inter-subject correlation values.

|  | 22:00 | | 23:00 | | 00:00 | | 01:00 | | 02:00 | | 03:00 | | 04:00 | | 05:00 | | 06:00 | | 07:00 | |
| --- | --- | --- | --- | --- | --- | --- | --- | --- | --- | --- | --- | --- | --- | --- | --- | --- | --- | --- | --- | --- |
| HR | $t\left( 46 \right)=-6.41$, $p<.001$ | | $t\left( 47 \right)=-3.19$, $p=.003$ | | $t\left( 46 \right)=-11.14$ $p<.001$ | | $t\left( 47 \right)=-8.31$, $p<.001$ | | $t\left( 46 \right)=-3.10$, $p=.003$ | | $t\left( 46 \right)=-9.36$, $p<.001$ | | $t\left( 46 \right)=-7.99$, $p<.001$ | | $t\left( 46 \right)=-2.79$, $p=.008$ | | $t\left( 45 \right)=-3.37$, $p=.002$ | | $t\left( 46 \right)=-5.71$, $p<.001$ | |
| EDA | | $t\left( 49 \right)=-3.77$,$p=.004$ | | $t\left( 50 \right)=-3.26$, $p=.002$ | | $t\left( 51 \right)=-3.86$, $p<.001$ | | $t\left( 51 \right)=-2.90$, $p=.005$ | | $t\left( 51 \right)=-0.15$, $p=.882$ | | $t\left( 50 \right)=-2.07$, $p=.043$ | | $t\left( 50 \right)=-0.44$, $p=.663$ | | $t\left( 50 \right)=-0.12$, $p=.906$ | | $t\left( 48 \right)=-1.41$, $p=.164$ | | $t\left( 49 \right)=-5.51$, $p<.001$ |

Supplementary Table 3. Statistical parameters of the two hierarchical linear models using permuted inter-subject correlations in either EDA (ISC-EDA) or heart rate (ISC-HR) as predictor of the number of correct answers about the content of the videos.

| Step | Predictor | -2LL | DF | AIC | $\boldsymbol{\chi}^{\boldsymbol{2}}$ -2LL change | R^2^ | $\boldsymbol{t}$ fixed effect |
| --- | --- | --- | --- | --- | --- | --- | --- |
| 1 |  | -804.78 | 2 | 1613.6 |  |  |  |
| 2 |  | -804.78 | 3 | 1615.6 | $\left( 1,N=446 \right)=0$, $p=1$ | 0 | 114*** |
| 3a | ISC-EDA | -804.3 | 4 | 1616.6 | $\left( 1,N=446 \right)=0.976$, $p=.323$ | .002 | -0.99 |
| 3b | ISC-HR | -803.68 | 4 | 1615.4 | $\left( 1,N=446 \right)=2.20$, $p=.138$ | .005 | -1.49 |
| 4a | ISC-EDA | -796.36 | 6 | 1604.7 | $\left( 2,N=446 \right)=15.87$, $p<.001$ | .068 | 0.31 |
| 4b | ISC-HR | -795.14 | 6 | 1602.3 | $\left( 2,N=444 \right)=17.08$, $p<.001$ | .075 | -1.85 |

* $p<.05$, ** $p<.01$, *** $p<.001$
